# Nuclear–Cytoplasmic Axis in Cancer: From Protein Mislocalization to Anticancer Drug Resistance

**DOI:** 10.32604/or.2026.083902

**Published:** 2026-08-13

**Authors:** Xueping Zhu, Misi He, Ling Wang, Rui Su, Lin Zhong, Ting Guo, Haixia Wang, Dongling Zou

**Affiliations:** 1 Department of Gynecologic Oncology, Chongqing University Cancer Hospital, Chongqing Cancer Institute, Chongqing Cancer Hospital, Chongqing, China; 2 Chongqing Specialized Medical Research Center of Ovarian Cancer, Chongqing, China; 3 Organoid Transformational Research Center, Chongqing Key Laboratory of Translational Research for Cancer Metastasis and Individualized Treatment, Chongqing University Cancer Hospital, Chongqing, China; 4 Department of Systems Biology, Beckman Research Institute of City of Hope, Monrovia, CA, USA

**Keywords:** Nucleocytoplasmic transport, nuclear pore complex, transport receptors, anticancer drug resistance, post-translational modifications, tumor microenvironment, nuclear localization signal mutations, nuclear export signal mutations

## Abstract

Nucleocytoplasmic transport (NCT) regulates the spatial distribution of proteins and RNA between the nucleus and cytoplasm. NCT dysregulation can mislocalize tumor suppressors, DNA-repair factors, transcription factors, and drug targets in cancer. In this review, we conceptualize NCT-dependent protein mislocalization as a spatial regulatory framework for anticancer drug resistance, rather than as a catalogue of transport components. We systematically discuss how nuclear pore complex (NPC) remodeling, transport-receptor imbalance, post-translational modification (PTM)-regulated cargo routing, signaling-NCT crosstalk, nuclear localization signal/nuclear export signal (NLS/NES) alterations, and tumor microenvironmental pressures jointly drive aberrant nucleocytoplasmic distribution. These processes can further regulate apoptosis, DNA-damage repair, oncogenic transcription, oxidative stress adaptation and drug-target accessibility, which ultimately promote drug tolerance and therapeutic resistance. We further distinguish clinically validated mechanisms from preclinical phenotypes and correlative observations. At present, the most advanced therapeutic evidence mainly supports exportin 1/chromosome region maintenance 1 (XPO1/CRM1) inhibition, particularly selinexor in selected hematologic malignancies; in contrast, strategies targeting the NPC, importins, PTM pathways, microenvironmental cues, or localization signals remain largely investigational. By integrating mechanistic, preclinical, translational, and clinical evidence, this review aims to synthesize current evidence on NCT-dependent protein mislocalization as a resistance-relevant axis and to highlight the need for cargo-specific biomarkers and rational combination strategies to translate this biology into anticancer therapy.

## 1 Introduction

Nucleocytoplasmic transport (NCT) controls the selective distribution of proteins and RNA between the nucleus and cytoplasm through the nuclear pore complex (NPC), karyopherins, Ran-GTPase cycling, and cargo-specific nuclear localization or export signals [1,2]. In cancer, this spatial control can be rewired causing tumor suppressors such as p53 [3], breast cancer susceptibility gene 1 (BRCA1) [4], and forkhead box O (FOXO) family proteins [5], oncogenic transcription factors such as nuclear factor kappa B (NF-κB) [6], and drug targets such as epidermal growth factor receptor (EGFR) [7] to undergo altered localization or trafficking, leading to diminished apoptosis, enhanced DNA-damage tolerance, and multidrug resistance [8,9].

Importantly, NCT is not an isolated transport process. It is dynamically regulated by post-translational modifications (PTMs) [10,11], oncogenic signaling cascades [10], and microenvironmental stresses, including hypoxia [12,13], nutrient starvation [14], inflammation [15], and mechanical stress [16]. These upstream inputs can expose or mask nuclear localization signal (NLS) and nuclear export signal (NES) motifs, alter transport-receptor engagement, and produce adaptive localization states during therapy [17].

Clinically, inhibition of nuclear export via exportin 1/chromosome region maintenance 1 (XPO1/CRM1) blockade, exemplified by Selinexor [18], the first-in-class selective inhibitor of nuclear export (SINE), has established NCT as a druggable axis in hematologic malignancies [19]. Evidence in solid tumors, including hepatocellular carcinoma (HCC)- and Kirsten rat sarcoma viral oncogene homolog (KRAS)-driven contexts, remains more heterogeneous and often preclinical or early clinical [20,21]. Therefore, clinical translation requires careful attention to tumor type, toxicity, cargo dependency, and predictive biomarkers [22,23].

Here, we synthesize NCT-associated protein mislocalization as a spatial regulatory framework linking aberrant cargo distribution to therapy response, rather than as a catalogue of transport components. We first trace how NPC remodeling, transport-receptor imbalance, PTM-regulated cargo routing, signaling crosstalk, localization-signal alterations, and tumor microenvironment (TME) pressures converge on aberrant nuclear-cytoplasmic distribution. We then distinguish clinically validated findings from preclinical and correlative evidence, with particular attention to the greater clinical maturity of XPO1 inhibition compared with other emerging NCT-directed strategies. By integrating mechanistic studies, preclinical models, clinical associations, and therapeutic trials, this review highlights both the mechanistic logic and the translational limits of targeting NCT in refractory cancers.

## 2 Historical Evolution of NCT Research: From Nuclear Transport Signals to Therapy Response

The modern concept of nucleocytoplasmic transport emerged from efforts to understand how selected proteins enter the nucleus rather than passively equilibrating across the nuclear envelope. A landmark step was the identification of the simian virus 40 (SV40) large T-antigen nuclear localization signal (NLS), which demonstrated that a short amino-acid sequence could be sufficient to direct a cytoplasmic protein into the nucleus, establishing localization signals as experimentally tractable determinants of subcellular distribution [24]. Subsequent identification of importin alpha/importin beta and the Ran-GTPase cycle defined the core machinery that interprets these signals and gives nuclear transport its directionality [25,26].

A second shift linked transport machinery to cancer-relevant cargo localization. CRM1/XPO1 was identified as a receptor for leucine-rich nuclear export signals, making nuclear export a defined receptor-mediated process rather than nonspecific leakage through the NPC [27]. This discovery later became especially relevant to oncology because many XPO1 cargoes include tumor suppressors, cell-cycle regulators, and stress-response proteins whose cytoplasmic displacement can weaken anticancer responses [28]. In parallel, studies of p53, BRCA1, and related regulators showed that altered subcellular localization can impair tumor-suppressive transcriptional programs or DNA-repair functions even when the protein is not simply absent [29,30]. These findings transformed protein mislocalization from a descriptive phenotype into a mechanistic route by which cancer cells may alter therapy response.

The current phase is increasingly translational and systems oriented. XPO1 inhibition, especially selinexor, has provided the clearest clinical proof that NCT can be pharmacologically targeted, with strongest evidence in selected hematologic malignancies [22,23]. However, recent work has shifted attention toward cargo-specific dependency, solid-tumor resistance, noncanonical XPO1 functions, and NPC- or nucleoporin-centered mechanisms involving chromatin regulation, nuclear mechanics, and selective-barrier dynamics [2,31,32,33,34]. Thus, NCT research has progressed from localization signals and transport machinery to cancer-associated cargo mislocalization and context-specific therapeutic targeting, which is the organizing logic of this review.

## 3 NCT Drives Resistance

### 3.1 NPC Dysfunction

During interphase, the NPC serves as a selective transport interface that helps maintain compartment-specific distribution of proteins and RNA between the nucleus and cytoplasm [35]. In cancer, remodeling of NPC composition, permeability, or chromatin-associated functions can shift cargo selectivity, allowing oncogenic factors to accumulate in the nucleus while tumor suppressors or DNA-repair regulators become mislocalized [36].

Malignancy-associated NPC changes first reshape the influx of survival-adaptive transcription factors. In prostate cancer, POM121 enhances NPC-mediated, importin-β-dependent nuclear import of E2F transcription factor 1 (E2F1), MYC proto-oncogene (MYC), and androgen receptor (AR), thereby reinforcing transcriptional programs that support tumor progression and resistance-associated phenotypes [37]. A related mechanism has been described in squamous malignancies, where the NUP62-KPNB1-ΔNp63 axis sustains nuclear import of ΔNp63α, represses the pro-apoptotic factor p53 upregulated modulator of apoptosis (PUMA), and promotes radioresistance [38].

NPC dysfunction can also impair nuclear retention or appropriate redistribution of tumor suppressors and DNA-repair mediators. Nucleoporin 153 (NUP153), for example, supports p53-binding protein 1 (53BP1) nuclear retention/import in daughter-cell nuclei; its loss compromises DNA double-strand break repair and alters clonogenic survival after ionizing radiation [39]. In neuroblastoma, the TSPYL5-G3BP1-RanBP2 axis links the NPC-associated factor Ran-binding protein 2/nucleoporin 358 (RanBP2/NUP358) to p53 SUMOylation, accelerated p53 nuclear export, reduced nuclear transcriptional activity, and cisplatin resistance [40].

Beyond classical pore-mediated import and export, nucleoporin-associated oncogenic programs may arise through chromatin-linked rewiring of transport junctions. In acute myeloid leukemia (AML), DEK::NUP214 behaves as an XPO1-dependent transcriptional activator at FOXC1 and HOXA/B regulatory loci, creating a chromatin-linked dependency coupled to therapeutic vulnerability [41]. In T-cell acute lymphoblastic leukemia (T-ALL), chromatin-bound CRM1 recruits SET::NUP214 to HOX clusters and enforces aberrant HOX transcriptional programs associated with corticosteroid and chemotherapy resistance [42]. In metastatic prostate cancer, off-pore sPOM121 cooperates with SMARCA5 at promoter-associated nuclear condensates to amplify β-catenin-centered transcriptional circuits, promoting therapy resistance and immune evasion [43].

Beyond these established routes, phase-separation properties of nucleoporins have emerged as a potential integrative layer of NPC dysfunction. Phenylalanine-glycine-rich (FG)-nucleoporins form a selective permeability barrier within the NPC central channel, and their phase behavior can influence how macromolecules and transport receptors partition through the pore [44]. In hematologic malignancy models, this concept also connects with chromatin-linked nucleoporin dysfunction, as nucleoporin 98 (NUP98) fusion proteins have been shown to form phase-separated condensates that drive aberrant chromatin looping, leukemogenic transcriptional programs, and malignant transformation [45,46]. However, direct evidence linking FG-nucleoporin phase behavior to therapy resistance remains limited. We therefore discuss liquid-liquid phase separation (LLPS)-related NPC dysfunction as an emerging hypothesis that may connect pore selectivity, chromatin-associated nucleoporin functions, and stress-induced cargo mislocalization, but requires cancer-specific functional validation.

**Figure 1:**
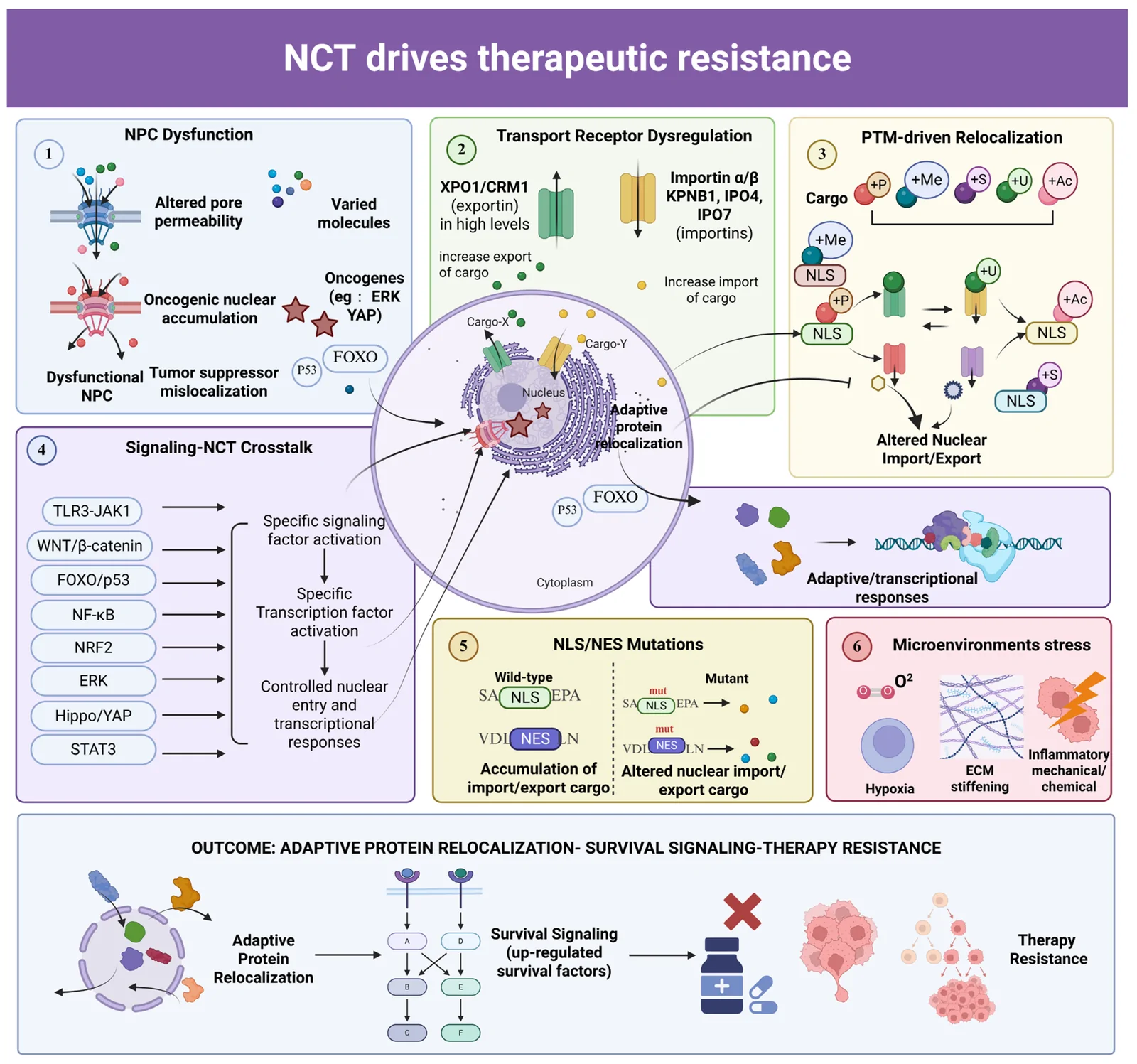
NCT dysregulation shapes anticancer drug-resistance phenotypes. Aberrant NCT can influence therapy response through six interconnected mechanisms. (1) NPC dysfunction: remodeling of the nuclear pore complex (NPC) alters pore permeability, resulting in nuclear accumulation of oncogenic factors and mislocalization of tumor suppressors. (2) Transport receptor dysregulation: abnormal activity of exportins and importins enhances nuclear export or import of resistance-related cargoes, thereby sustaining pro-survival signaling. (3) PTM-driven relocalization: post-translational modifications modulate the exposure of nuclear localization or export signals and reprogram cargo trafficking between the nucleus and cytoplasm. (4) Signaling-NCT crosstalk: oncogenic signaling pathways hijack NCT to control the localization and transcriptional activity of key downstream effectors. (5) NLS/NES mutations: alterations in nuclear localization or export motifs redirect subcellular trafficking and reshape drug responses. (6) Microenvironmental stress: hypoxia, extracellular matrix stiffening, and inflammatory, mechanical, or chemical stress induce adaptive protein relocalization through NCT dysregulation. Collectively, these mechanisms converge on adaptive protein relocalization, enhanced survival signaling, impaired apoptosis, and resistance-associated phenotypes, with clinical strength varying by cargo, tumor type, and therapeutic context. The figure was prepared using BioRender.com.

Collectively, these examples show that NPC dysfunction contributes to resistance-associated phenotypes through at least three routes: altered transcription-factor influx, defective tumor-suppressor or DNA-repair cargo localization, and chromatin-linked transport rewiring. LLPS-related nucleoporin biology further expands this framework by suggesting how NPC selective permeability and chromatin-associated nucleoporin functions may be coupled to cancer-relevant transcriptional states, although direct evidence connecting this layer to therapy resistance remains limited. The strongest mechanistic evidence comes from defined cargo-specific models, whereas the broader clinical value of NPC alterations will require tumor-type-specific validation and biomarker development (Table 1 & Fig. 1).

**Table 1:** Nuclear pore complex (NPC) dysfunction contributes to therapy resistance through distinct mechanistic routes.

Mechanistic Category	NPC Component(s)/Factor	Cargo/Pathway Affected	Cancer Type/Model	Mechanism of Resistance	Ref.
Increased influx of survival-adaptive transcription factors	POM121	E2F1, MYC, AR (importin-β–dependent)	Prostate cancer (cell lines, PDX, mouse models)	NPC-mediated nuclear import enhances oncogenic transcription programs and tumor progression	[37]
	NUP62	ΔNp63α (importin-β–dependent)	HNSCC (SCC models)	Facilitates ΔNp63 nuclear entry, suppresses PUMA, supports radioresistance	[38]
Nuclear retention defects of tumor suppressors	NUP153	53BP1	Human cell models	Loss impairs nuclear retention/import of 53BP1 in daughter nuclei, suppresses DSB repair	[39]
	RanBP2 (NUP358), TSPYL5–G3BP1 apparatus	p53 (SUMOylation-mediated ejection)	Neuroblastoma	Promotes p53 SUMOylation and nuclear export, diminishing nuclear p53 activity	[40]
Chromatin-linked transport junction rewiring	DEK::NUP214, XPO1	FOXC1 and HOXA/B transcriptional loci	AML	DEK::NUP214 behaves as an XPO1-dependent transcriptional activator at FOXC1 and HOXA/B regulatory loci, establishing a chromatin-linked dependency coupled to therapeutic vulnerability	[41]
	SET::NUP214, CRM1/XPO1	HOX transcriptional program	T-ALL	Chromatin-bound CRM1 recruits SET::NUP214 to HOX clusters, enforcing aberrant HOX transcriptional programs associated with corticosteroid and chemotherapy resistance	[42]
	off-pore sPOM121, SMARCA5	β-catenin-centered transcriptional circuitry	Metastatic prostate cancer	sPOM121 cooperates with SMARCA5 at promoter-associated nuclear condensates to amplify β-catenin-centered transcriptional programs, thereby promoting therapy resistance and immune evasion	[43]

Abbreviations: NPC, nuclear pore complex; DSB, DNA double-strand break; HNSCC, head and neck squamous cell carcinoma; AML, acute myeloid leukemia; T-ALL, T-cell acute lymphoblastic leukemia.

### 3.2 Transport Receptor Dysregulation

Export and import transport receptors are principal exponents of NCT, and dysregulation of those receptors is another widespread mode of resistance [1,31]. More specifically, the export carrier XPO1/CRM1 has emerged as a chief mediator [47,48]. Overexpression of XPO1 results in aberrant cytoplasmic exclusion of tumor suppressors such as p53 [49], and FOXO [50], effectively silencing their transcriptional agendas and bringing about drug resistance [32,51,52].

Importins are also involved. For instance, importin α/β-dependent nuclear entry of survival inhibitors such as X-linked inhibitor of apoptosis protein (XIAP) underlies resistant phenotypes, and suppression of importin α/β reduces survival under drug pressure [53]. Moreover, in castration-resistant prostate cancer, importin-β1-dependent nuclear import of androgen receptor-V7 (AR-V7) and related drivers sustains ligand-independent AR signaling, thereby contributing to enzalutamide resistance [54]. Similarly, in cervical cancer, karyopherin subunit beta 1 (KPNB1)/importin β1-mediated nuclear import contributes to cisplatin resistance by restraining p53 stabilization and activation while facilitating NF-κB nuclear translocation [55]. Consistently, aberrant importin β1-dependent nuclear influx represents a mechanistically relevant contributor to chemoresistance, highlighting dysregulated nuclear import as a resistance-promoting pathway [56]. Furthermore, importin-4 (IPO4) promotes cisplatin resistance in cervical cancer by conveying CCAAT/enhancer-binding protein delta (CEBPD) to the nucleus, whereby CEBPD activates protein kinase, DNA-activated, catalytic subunit (PRKDC; DNA-dependent protein kinase catalytic subunit, DNA-PKcs)–mediated DNA repair, opening up the potential of targeting nuclear influx pathways to overcome chemotherapy resistance [57]. Finally, importin-7-dependent nuclear influx of AR and its counterpart counterpart ubiquitin-specific peptidase 22 (USP22) retains AR-dependent transcription in breast cancer, reducing AR antagonist efficacy [58]. Key pathways are presented in Table 2 & Fig. 1.

**Table 2:** Representative exportin/importin receptor-mediated mechanisms of anticancer drug resistance.

Exportin/Importin	Cargo/Target	Cancer Type/Model	Mechanism of Resistance	Ref.
XPO1/CRM1	p53, FOXO	Multiple cancers/pan-cancer context	XPO1 overexpression drives aberrant cytoplasmic sequestration of tumor suppressors such as p53 and FOXO, thereby silencing their transcriptional programs and promoting drug resistance.	[47,48,49,50,51,52]
Importin α/β	XIAP	Drug-stressed cancer models	Importin α/β-dependent nuclear import of XIAP supports cell survival under therapeutic stress and underlies resistant phenotypes.	[53]
Importin-β1 (KPNB1)	AR-V7 and related AR drivers	Castration-resistant prostate cancer	Importin-β1-dependent nuclear import of AR-V7 and related drivers sustains ligand-independent AR signaling, thereby contributing to enzalutamide resistance.	[54]
	p53, NF-κB	Cervical cancer	KPNB1/importin β1-mediated nuclear import promotes cisplatin resistance by restraining p53 stabilization and activation while facilitating NF-κB nuclear translocation.	[55]
	Multiple oncogenic cargos	Multiple models	Aberrant importin β1-dependent nuclear influx represents a general resistance-promoting pathway and contributes to chemoresistance.	[56]
Importin-4 (IPO4)	CEBPD–PRKDC (DNA-PKcs) axis	Cervical cancer	IPO4-mediated nuclear translocation of CEBPD activates PRKDC-mediated DNA repair, thereby promoting cisplatin resistance.	[57]
Importin-7 (IPO7)	AR, USP22	Breast cancer	Importin-7-dependent nuclear import of AR and USP22 sustains AR-dependent transcription and reduces the efficacy of AR antagonists.	[58]

Abbreviations: NCT, nucleocytoplasmic transport; XPO1/CRM1, exportin 1; XIAP, X-linked inhibitor of apoptosis protein; AR-V7, androgen receptor splice variant 7; KPNB1, karyopherin β1; NF-κB, nuclear factor kappa-B; IPO4, importin-4; CEBPD, CCAAT/enhancer-binding protein delta; PRKDC/DNA-PKcs, DNA-dependent protein kinase catalytic subunit; USP22, ubiquitin-specific peptidase 22; AR, androgen receptor.

### 3.3 Post-Translational Modifications (PTMs)

Post-translational modifications (PTMs) have important effects on protein activity and localization, influencing NCT and therefore drug resistance [17]. In this section, PTMs are discussed as molecular switches that directly alter cargo trafficking by exposing or masking NLS/NES motifs, changing cargo affinity for importins or exportins, or modifying nuclear/cytoplasmic retention. Because many PTMs are induced by oncogenic or stress-activated signaling, this section focuses on the modification-level mechanism, whereas pathway-level crosstalk is discussed in Section 3.4.

#### 3.3.1 Methylation

Methylation has been identified as a dynamic regulator of NCT with potential relevance to therapy response. By changing the localization, stability, or interaction partners of modified substrates, methylation can alter nuclear retention, import, or export and thereby reshape the activity of oncogenic effectors, tumor suppressors, and transcription factors [59,60].

One of the representative direct mechanisms is PRMT6-mediated methylation of p21 at Arg156, augmenting its cytoplasmic localization, abolishing its nuclear cell-cycle checkpoint activity, and hence rendering cancer cells tolerant to drugs [61]. It defines a bona fide pathway from localization alteration by methylation to resistance to drugs.

Recent advances extend the paradigm to oncogenic transcriptional regulators. More recently, methylation of Yes-associated protein (YAP) at Arg124 by protein arginine methyltransferase 1 (PRMT1) was shown to generate a positive feedback loop with the methionine transporter solute carrier family 43 member 2 (SLC43A2) and thus maintain YAP activity and resistance to anticancer drugs [62]. This example supports methylation-directed relocalization as a resistance-relevant NCT mechanism.

#### 3.3.2 Phosphorylation

Phosphorylation has emerged as a critical post-translational switch governing subcellular localization and therapy response [63,64]. For instance, tyrosine phosphorylation of signal transducer and activator of transcription 3 (STAT3) results in its nuclear translocation and transcriptional activation of survival genes, maintaining chemoresistance and radioresistance in many cancers [63]. In colorectal cancer, BRAF-mediated phosphorylation of KIAA1429 drives its cytoplasmic redistribution, stabilizing frizzled class receptor 7 (FZD7) and activating Wingless/INT-1 (WNT) signaling to induce oxaliplatin resistance [65]. In ovarian cancer, NIMA-related kinase 6 (NEK6) phosphorylates FOXO3 at Ser7, preventing nuclear entry, stabilizing c-MYC signaling, and conferring platinum resistance [66]. Similarly, Janus kinase 1 (JAK1)-mediated phosphorylation of toll-like receptor 3 (TLR3) at Ser155 enhances its importin-α5-dependent nuclear import, driving c-MYC-linked chemoresistance and metastasis [67]. Expanding this paradigm, p38 mitogen-activated protein kinase (p38-MAPK) phosphorylates glutamate-cysteine ligase modifier subunit (GCLM) at Thr17 in colorectal cancer, promoting importin-α5 interaction, nuclear accumulation, and platinum resistance through NF-κB-repressing factor (NKRF) regulation [68]. In triple-negative breast cancer, cyclin-dependent kinase 2 (CDK2)-dependent phosphorylation facilitates tripartite motif-containing protein 32 (TRIM32) nuclear entry, stabilizing nuclear pSTAT3 and driving radioresistance [69]. Additional examples further highlight this mechanism: in epidermal growth factor receptor (EGFR)-mutant non-small cell lung cancer (NSCLC), p21-activated kinase 2 (PAK2) phosphorylates β-catenin to promote nuclear accumulation and osimertinib resistance [70]; in ovarian cancer, PDZ-binding kinase (PBK) phosphorylates tripartite motif-containing protein 37 (TRIM37), inducing NF-κB activation and poly(ADP-ribose) polymerase (PARP) inhibitor resistance [71]. In hormone receptor-positive breast cancer, serum/glucocorticoid-regulated kinase 3 (SGK3) phosphorylates glycogen synthase kinase 3 beta (GSK3β) and β-catenin, reinforcing nuclear β-catenin enrichment and resistance to phosphoinositide 3-kinase alpha (PI3Kα) inhibitors, which is reversed by SGK3 blockade [72]. These illustrative cases indicate that phosphorylation is an active modulator of NCT and highlight its role in chemoresistance phenotype determination.

#### 3.3.3 SUMOylation

Small ubiquitin-like modifier (SUMO)ylation is crucial for protein localization and stability, thus influencing therapeutic response [73]. Aberrant SUMOylation may alter subcellular partitioning of target proteins, leading to acquired drug resistance. An example is incubation with the TOP1 inhibitor camptothecin, resulting in SUMOylation of Topoisomerase I (TOP1) that alters its partitioning between nucleoplasm and nucleoli to influence availability of TOP1 and confer acquired resistance to camptothecin [74]. Secondly, in cholangiocarcinoma, hyper-SUMOylation of p27^Kip1 enhances its export from the nucleus and retention in cytoplasm, impairing its cell-cycle inhibitory activity and promoting cisplatin resistance [75]. Similarly, in pancreatic ductal adenocarcinoma (PDAC), cancer-associated fibroblast–derived circBIRC6 enhances SUMOylation of X-ray repair cross-complementing protein 4 (XRCC4) at K115, promoting its chromatin recruitment and nuclear accumulation, which strengthens non-homologous end joining (NHEJ) repair and drives oxaliplatin resistance; genetic disruption of XRCC4 SUMOylation (K115R) or pharmacological inhibition with 2-D08 re-sensitizes tumors to platinum and further synergizes with PARP inhibitors in xenograft models [76]. These results demonstrate that SUMO-regulated protein trafficking is a significant modality of chemotherapy evasion.

#### 3.3.4 Ubiquitination

Ubiquitination dynamically modulates protein stability and trafficking, thereby influencing therapy response [77]. Recent studies have uncovered mechanisms by which ubiquitination-dependent relocalization of oncogenic regulators feeds directly into therapy resistance. In glioma, the tripartite motif-containing protein 25 (TRIM25) E3 ligase promotes Kelch-like ECH-associated protein 1 (Keap1) ubiquitination, thereby enhancing erythroid 2-related factor 2 (NRF2) nuclear import/retention and activating antioxidant programs that support temozolomide resistance [78]. This finding points out ubiquitination as an essential post-translational switch that remodels the subcellular localization of key effectors and provides an adaptive foundation for drug resistance.

#### 3.3.5 Glycosylation

Glycosylation, particularly N-glycosylation, is important for the localization and activity of chemoresistance-conferring membrane proteins [79,80]. In docetaxel-resistant Michigan Cancer Foundation-7/adriamycin-resistant (MCF7-ADR) breast cancer cells, ribophorin II (RPN2)-dependent glycoslation of P-glycoprotein (P-gp) is required for its correct membrane localization. RPN2 silencing reduces P-gp glycosylation and membrane expression, restoring docetaxel sensitivity *in vitro* and *in vivo* [81]. In addition, O-GlcNAc transferase (OGT)-mediated O-GlcNAcylation of microphthalmia-associated transcription factor (MITF) at S49 within its NLS enhances its binding to importin α/β, thereby promoting MITF nuclear translocation and contributing to Cyclin-dependent kinases 4 and 6 (CDK4/6) inhibitor resistance in breast cancer [82]. These findings illustrate how glycosylation-induced subcellular localization changes can contribute to chemoresistance.

#### 3.3.6 Acetylation

Acetylation has emerged as a direct modulator of nucleocytoplasmic transport with important therapy-response implications. One representative example is that of N-acetyltransferase 10 (NAT10)-dependent acetylation of ATP-citrate lyase (ACLY) at lysine 468, which promotes ACLY nuclear translocation under chemotherapeutic stress. Nuclear ACLY increases nuclear acetyl-CoA synthesis, thereby supporting histone acetylation and gene transcription of drug-resistance genes such as cytochrome P450 family 2 subfamily C member 9 (CYP2C9) and phosphoinositide-3-kinase regulatory subunit 1 (PIK3R1). Functionally, this pathway confers heightened chemoresistance in hepatocellular carcinoma models [83]. This example establishes a causal link from acetylation-dependent subcellular relocalization to drug resistance, and positions acetylation not only as a modulator of both metabolism and NCT-dependent therapy response.

PTMs constitute a dynamic regulatory layer that connects NCT with drug resistance, all above mechanisms have shown in the Table 3 & Fig. 1. Evidence shows that PTMs such as methylation, phosphorylation, SUMOylation, ubiquitination, acetylation, and glycosylation can reset nuclear import, export, or retention of central regulators, including transcription factors, tumor suppressors, and DNA-repair proteins [84]. From a translational standpoint, PTMs create both vulnerabilities and therapeutic opportunities. Inhibitors of PRMTs [85], HDACs [86], or PARP enzymes [87] are approved entities for clinical use or are being evaluated in clinical trials, but their implications for NCT-dependent resistance remain insufficiently characterized [17]. Key gaps include tumor-type specificity, PTM crosstalk, and predictive biomarkers that identify patients most likely to benefit from NCT-directed combinations.

**Table 3:** PTM-mediated regulation of nucleocytoplasmic transport and therapy resistance.

PTM Type	Protein/Target	Modification & NCT Effect	Cancer Context	Ref.
Methylation	p21	PRMT6-mediated methylation at Arg156 promotes cytoplasmic relocalization of p21, abolishing its nuclear cell-cycle checkpoint activity and thereby conferring drug tolerance.	Cancer cells	[61]
	YAP	PRMT1-mediated methylation at Arg124 sustains YAP activity through relocalization-associated positive feedback with SLC43A2, thereby promoting resistance to anticancer drugs.	Cancer	[62]
Phosphorylation	STAT3	Tyrosine phosphorylation induces STAT3 nuclear translocation and activation of survival genes, thereby maintaining chemo- and radioresistance.	Multiple cancers	[63]
	KIAA1429	BRAF-mediated phosphorylation drives cytoplasmic redistribution of KIAA1429, stabilizing FZD7 and activating WNT signaling to promote oxaliplatin resistance.	Colorectal cancer	[65]
	FOXO3	NEK6-mediated phosphorylation at Ser7 prevents FOXO3 nuclear entry, stabilizes c-MYC signaling, and confers platinum resistance.	Ovarian cancer	[66]
	TLR3	JAK1-mediated phosphorylation at Ser155 enhances importin-α5-dependent nuclear import of TLR3, driving c-MYC-linked chemoresistance and metastasis.	Cancer	[67]
	GCLM	p38-MAPK-mediated phosphorylation at Thr17 promotes importin-α5 interaction and nuclear accumulation of GCLM, contributing to platinum resistance through NKRF regulation.	Colorectal cancer	[68]
	TRIM32	CDK2-dependent phosphorylation facilitates TRIM32 nuclear entry and stabilizes nuclear pSTAT3, thereby driving radioresistance.	Triple-negative breast cancer	[69]
	β-catenin	PAK2-mediated phosphorylation promotes nuclear accumulation of β-catenin and contributes to osimertinib resistance.	EGFR-mutant NSCLC	[70]
	TRIM37	PBK-mediated phosphorylation induces NF-κB activation and promotes PARP inhibitor resistance.	Ovarian cancer	[71]
	GSK3β/β-catenin	SGK3-mediated phosphorylation reinforces nuclear β-catenin enrichment, thereby promoting resistance to PI3Kα inhibitors.	Hormone receptor-positive breast cancer	[72]
SUMOylation	TOP1	Camptothecin-induced SUMOylation alters TOP1 partitioning between nucleoplasm and nucleoli, reducing effective TOP1 availability and conferring acquired resistance.	Cancer cells	[74]
	p27^Kip1	Hyper-SUMOylation enhances nuclear export and cytoplasmic retention of p27^Kip1, impairing its cell-cycle inhibitory function and promoting cisplatin resistance.	Cholangiocarcinoma	[75]
	XRCC4	circBIRC6-driven SUMOylation of XRCC4 at K115 promotes chromatin recruitment and nuclear accumulation of XRCC4, strengthening NHEJ repair and driving oxaliplatin resistance.	PDAC	[76]
Ubiquitination	Keap1/NRF2	TRIM25-mediated ubiquitination of Keap1 enhances nuclear import/retention of NRF2, activating antioxidant programs and promoting temozolomide resistance.	Glioma	[78]
Glycosylation	P-gp	RPN2-dependent N-glycosylation is required for proper membrane localization of P-gp; loss of this modification reduces membrane expression and restores docetaxel sensitivity.	Docetaxel-resistant breast cancer (MCF7-ADR)	[81]
	MITF	O-GlcNAcylation at Ser49 within the NLS enhances importin α/β binding and promotes MITF nuclear localization.	Breast cancer	[82]
Acetylation	ACLY	NAT10-dependent acetylation of ACLY at Lys468 promotes its nuclear translocation under chemotherapeutic stress, increasing nuclear acetyl-CoA production, histone acetylation, and expression of drug-resistance genes.	Hepatocellular carcinoma	[83]

Abbreviations: PTM, post-translational modification; NCT, nucleocytoplasmic transport; CRM1, chromosome region maintenance 1/exportin 1; YAP, Yes-associated protein; STAT3, signal transducer and activator of transcription 3; NSCLC, non-small cell lung cancer; TOP1, topoisomerase I; NHEJ, non-homologous end joining; PDAC, pancreatic ductal adenocarcinoma; NRF2, nuclear factor erythroid 2-related factor 2; EMT, epithelial-mesenchymal transition; P-gp, P-glycoprotein; MITF, microphthalmia-associated transcription factor; ACLY, ATP-citrate lyase.

### 3.4 Signaling Pathways and NCT Crosstalk in Resistance

Whereas Section 3.3 focuses on PTMs as molecular switches for individual cargo trafficking, this section discusses signaling pathways as broader regulatory contexts that coordinate NCT changes across multiple cargoes and resistance phenotypes.

Stress-response and DNA-damage pathways provide one major entry point into this crosstalk. In the TLR3–JAK1 axis, JAK1-dependent nuclear redistribution of TLR3 links stress signaling to c-MYC-associated chemoresistance and metastatic phenotypes [67]. In the extracellular signal-regulated kinase (ERK)–RFNG O-fucosylpeptide 3-beta-N-acetylglucosaminyltransferase (RFNG) axis, ERK-dependent RFNG nuclear translocation through the karyopherin subunit alpha 1/karyopherin subunit beta 1 (KPNA1/KPNB1) import pathway suppresses p53 activity and contributes to oxaliplatin resistance [88]. These examples illustrate how stress-linked kinase signaling can convert a localization change into altered apoptosis, repair, and chemotherapy response.

Survival and inflammatory pathways show a similar dependence on transcription-factor residency. In WNT/β-catenin signaling, β-catenin nuclear enrichment reinforces drug-tolerant transcriptional programs in BRAFV600E-mutant colorectal cancer, EGFR-mutant non-small cell lung cancer and PI3Kα inhibitor-resistant breast cancer models [65,70,72]. In the PI3K-AKT-FOXO axis, AKT-dependent FOXO nuclear exclusion limits FOXO-mediated apoptosis and cell-cycle checkpoint transcription, thereby shaping chemotherapy response [89,90]. NF-κB signaling illustrates how nuclear localization of either pathway regulators or upstream modulators can sustain resistant transcriptional output. In the PBK-TRIM37-NF-κB axis, PBK-driven TRIM37 nuclear translocation activates NF-κB signaling and promotes PARP inhibitor resistance in ovarian cancer [71]. In a therapeutically opposite direction, XPO1 inhibition can retain IκBα in the nucleus, suppress NF-κB signaling, and cooperate with proteasome inhibition [91]. In IL-6/STAT3 signaling, cytokine-induced p-STAT3 nuclear translocation drives an IL-6R/STAT3/miR-204 feedback loop that promotes cisplatin resistance in epithelial ovarian cancer cells [92].

Developmental, metabolic, and microenvironment-responsive pathways further illustrate how signaling inputs are translated into NCT-dependent resistance phenotypes. In Hippo/YAP signaling, regulated YAP/TAZ nuclear trafficking supports oncogenic transcriptional output; SOX9-driven YAP nuclear entry in hepatocellular carcinoma and hypoxia/HIF-1α-driven YAP nuclear entry can protect cancer cells from DNA-damage-induced apoptosis [93,94,95]. In NRF2-mediated redox signaling, nuclear NRF2 activity reinforces antioxidant defense and stress-adapted drug tolerance [78].

Together, these pathway-level patterns support NCT-mediated signaling rewiring as a convergent route to resistance-associated phenotypes. Importantly, the evidence strength varies by pathway: some mechanisms directly link a defined signaling event to cargo relocalization and drug response, whereas others remain pathway-level associations that require rescue experiments and clinical validation. Representative signaling-NCT axes are summarized in Table S1 & Fig. 1.

### 3.5 NLS/NES Mutations as an Evolutionary Strategy for Drug Resistance

Beyond post-translational regulation, a subset of somatic or engineered mutations that directly alter nuclear localization (NLS) or export signal (NES) motifs have been shown to reprogram subcellular routing and reshape therapeutic responses. In lung cancer, hotspot substitutions within the putative NES of EGFR (L747S/P) impair nuclear export and promote nuclear accumulation of EGFR, which in turn attenuates sensitivity to multiple EGFR tyrosine kinase inhibitors *in vitro* and in xenograft models [96].

Similarly, in etoposide-resistant small-cell lung cancer, a C-terminal truncation of Topoisomerase IIα (TOP2A) eliminates its tripartite NLS, driving cytoplasmic mislocalization that constitutes a distinct mechanism of resistance to topoisomerase II–targeted drugs [97]. Conversely, point mutations disrupting the NES of Survivin (BIRC5) abrogate CRM1-mediated export and trap the protein in the nucleus, thereby diminishing its anti-apoptotic activity and sensitizing tumor cells to radiation and temozolomide treatment [98]. Experimental ΔNLS constructs of p27^Kip1 produce cytoplasmic enrichment of the cyclin-dependent kinase inhibitor, blunting apoptosis and conferring tolerance to HER-family–targeted therapies in breast-cancer models [99]. Finally, studies employing engineered BRCA1 variants with forced nuclear localization (NLS-BRCA1) or cytoplasmic anchoring (NES-BRCA1) demonstrate that subcellular distribution alone can reprogram DNA-repair competence and modulate responsiveness to platinum compounds and PARP inhibitors [30].

Collectively, these examples provide direct mechanistic proof that sequence-level alterations of NLS/NES elements can redefine the intracellular routing of key regulators and thereby dictate drug sensitivity or resistance (Table S2 & Fig. 1).

### 3.6 Microenvironmental Pressures and NCT-Dependent Resistance

Accumulating evidence demonstrates that distinct TME cues can reprogram therapy responses by reshaping the NCT of key regulators. Among these stressors, hypoxia is the most extensively characterized. In hepatocellular carcinoma, hypoxic exposure drives YAP nuclear translocation and activation, thereby conferring resistance to SN38 [100]. Similarly, hypoxia-induced Twist1 expression promotes nuclear accumulation of EGFR and DNA-PKcs, enhancing DNA-repair capacity and radioresistance in cervical cancer, whereas blockade of this pathway re-sensitizes tumors to irradiation [101]. In colorectal cancer, hypoxia-triggered miR-675-5p expression stabilizes β-catenin nuclear localization, leading to 5-fluorouracil resistance [102]. In breast-cancer models, low oxygen activates nuclear NRF2 signaling, strengthening antioxidant defenses and reducing cisplatin efficacy [103].

Beyond oxygen deprivation, mechanical stiffening of the extracellular matrix (ECM) constitutes another potent TME determinant. Increased matrix rigidity induces nuclear enrichment of YAP/TAZ (and β-catenin) and promotes doxorubicin resistance in three-dimensional breast-cancer cultures, while similar stiffness-dependent nuclear YAP activation underlies gemcitabine tolerance in pancreatic-cancer models [104,105]. These findings indicate that physical properties of the TME can influence therapy response by altering the nuclear residency of mechanosensitive transcriptional regulators.

By contrast, lactate-rich or hypoxic milieus can trigger atypical nuclear trafficking events, such as GPR81 nuclear import, representing a nascent but intriguing form of TME-NCT interplay [106]. Because direct evidence linking lactate/GPR81 nuclear transport to therapy resistance remains limited, this axis is best presented as an emerging TME-NCT mechanism with resistance relevance still requiring validation.

Collectively, these findings establish a unifying model in which microenvironmental stresses, whether chemical, mechanical, or inflammatory, drive adaptive protein relocalization through NCT dysregulation, thereby enforcing drug-resistant phenotypes and unveiling actionable vulnerabilities within the nuclear-cytoplasmic transport machinery. Hypoxia-driven examples currently provide the strongest direct links between TME cues, NCT changes, and drug or radiation resistance, whereas stiffness- and lactate-associated mechanisms expand the framework to physical and metabolic TME inputs. Representative studies delineating the link between specific TME pressures, NCT alterations, and therapy resistance are systematically summarized in Table S3 & Fig. 1.

## 4 Therapeutic Targeting of NCT-Mediated Resistance

The mechanistic links described above suggest several therapeutic entry points, but these strategies differ markedly in translational maturity. XPO1 inhibition currently provides the strongest clinical validation, whereas importin targeting, NPC-directed intervention, PTM or signaling modulation, microenvironmental normalization, and localization-signal correction remain largely investigational or preclinical. Accordingly, this section evaluates NCT-targeted strategies according to both their intervention level and the maturity of supporting evidence.

**Figure 2:**
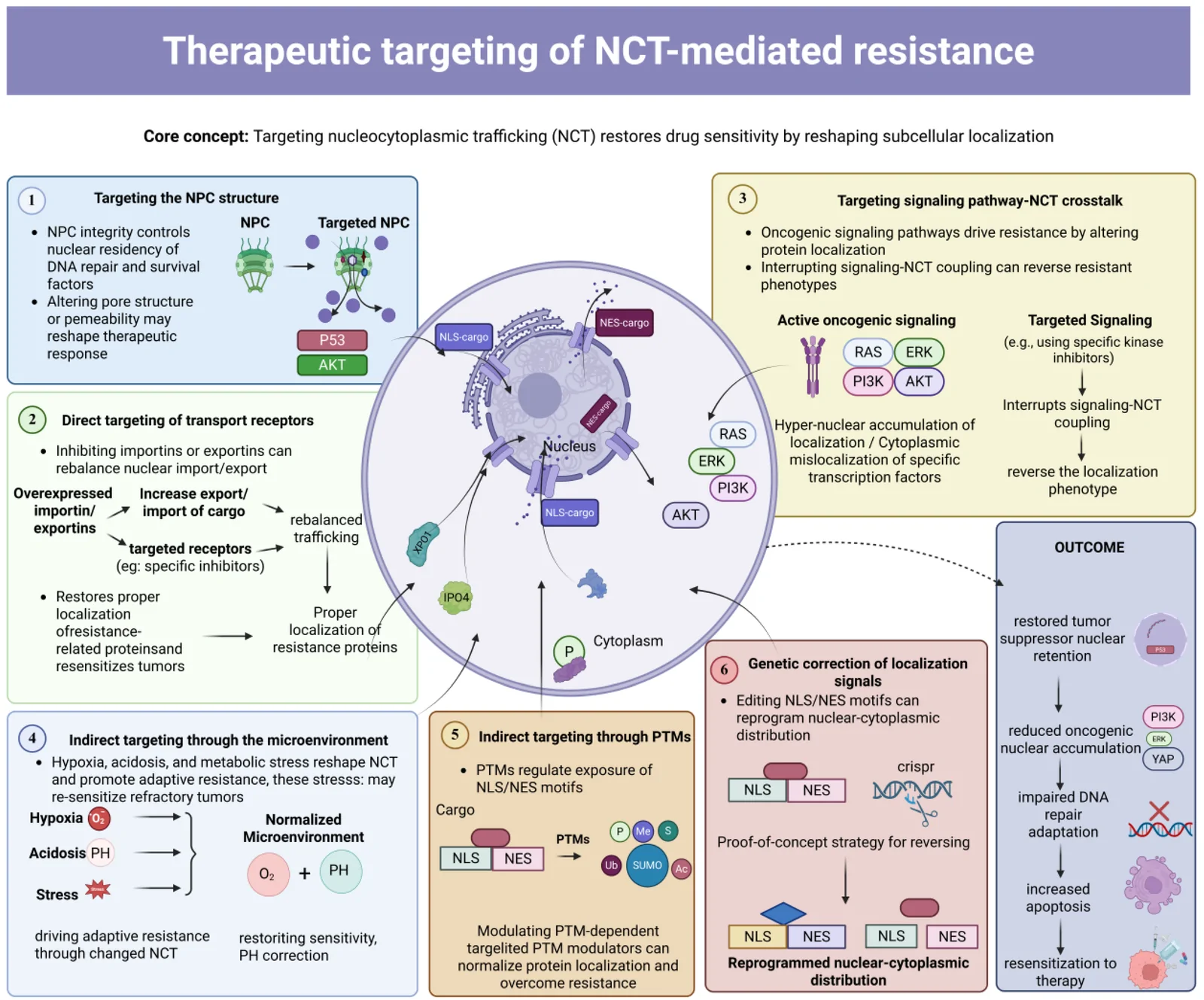
Therapeutic targeting of NCT-mediated resistance. The schematic illustrates six major therapeutic strategies designed to restore drug sensitivity in cancer cells by reshaping the subcellular localization of resistance-related proteins. (1) Targeting the NPC structure: Modulating nuclear pore complex (NPC) integrity and permeability to regulate the nuclear residency of DNA repair and survival factors. (2) Direct targeting of transport receptors: Utilizing specific inhibitors against overexpressed importins or exportins (e.g., XPO1, KPNB1) to rebalance nuclear import/export dynamics. (3) Targeting signaling pathway–NCT crosstalk: Applying targeted kinase inhibitors to disrupt oncogenic signaling cascades (e.g., RAS/ERK, PI3K/AKT) that drive the mislocalization of specific transcription factors. (4) Indirect targeting through the microenvironment: Normalizing tumor microenvironmental stresses, such as hypoxia and acidosis, to reverse adaptive resistance driven by altered NCT. (5) Indirect targeting through PTMs: Modulating targeted PTMs (phosphorylation, methylation, SUMOylation, ubiquitination, and acetylation) that regulate the exposure of NLS/NES motifs to normalize protein trafficking. (6) Genetic correction of localization signals: Utilizing genome-editing tools (e.g., CRISPR) to alter NLS/NES motifs, serving as a proof-of-concept strategy for reprogramming nuclear-cytoplasmic distribution. Final Outcome: Cumulatively, these synergistic interventions promote the nuclear retention of tumor suppressors (e.g., p53), attenuate the nuclear accumulation of oncogenes (e.g., ERK, YAP), impair DNA repair adaptations, and increase apoptosis, ultimately leading to the resensitization of cancer cells to therapy. The figure was prepared using BioRender.com.

Comprehensive research establishes that therapies targeting at different levels of the NCT machinery can restore chemosensitivity by reshaping the subcellular trafficking of resistance-related proteins [107]. These therapies can be stratified into six classes: direct targeting of transport receptors, direct targeting of NPC structure, indirect modulation through post-translational modifications, indirect modulation through signaling pathways, microenvironmental targeting, and genetic repair of localization determinants. Details are summarized in Table 4 & Fig. 2.

### 4.1 Targeting the NPC Structure

Beyond importins and exportins, nucleoporins themselves are assuming therapeutic node status. Building on prior evidence that NPC integrity governs the nuclear import of DNA-repair mediators such as 53BP1 [39] (see Section 3.1), subsequent functional studies have shown that nucleoporin dysregulation broadly influences DNA repair capacity and treatment outcomes across cancer types. In the clinic, high NUP153 expression has correlated with poor prognosis and reduced chemotherapy susceptiblitiy for various cancers [108]. *In vitro* disruption of NPC assembly selectively killed actively cycling tumor cells, further underscoring the therapeutic potential of selectively perturbing nuclear pore integrity [109]. Recent mechanistic work has expanded this concept to NPC functional permeability. In breast-cancer models, mechanical modulation of the nuclear envelope—via cytoskeletal tension and lamin–NPC coupling—was shown to alter pore gating and enhance nuclear accumulation of doxorubicin, restoring drug efficacy in previously resistant cells [33].

As direct evidence relating NPC rescue through drug resistance reversal still remains to be found, these studies consequently suggest that structural integrity of NPC dictates the nuclear residency of DNA repair and viability factors and hence therapeutic outcome.

Accordingly, NPC-directed intervention should currently be framed as an emerging preclinical concept rather than as a clinically validated resistance-reversal strategy; in this area, the key unmet need is to connect specific NPC alterations to drug response through functional rescue experiments and biomarker-defined tumor subsets [33,109].

Consistent with this view, accumulating clinical evidence indicates that NPC dysfunction actively drives aggressive malignant phenotypes rather than merely accompanying tumor progression. At present, no drugs directly act upon NPC structure per se. Drug advances instead stem from indirect interventions, most remarkably nuclear export inhibitors such as selinexor [110], which has been approved for multiple myeloma and has also been shown to restore chemosensitivity in preclinically relevant models. Looking ahead, it will be important to define the heterogeneity and functional consequences of NPC alterations across tumor contexts. Recent mechanistic evidence [111] linking nucleoporin dysregulation to aggressive tumor phenotypes further supports a cautious but actionable perspective: NPC-directed intervention remains preclinical, but tumor-context-specific nucleoporin alterations may help define biomarker-selected vulnerabilities for future therapeutic development.

### 4.2 Direct Targeting of Transport Receptors

Previous studies have already demonstrated that direct inhibition of import receptors such as Importin-4 [57] and Importin-β1 [55] can restore cisplatin sensitivity in cervical-cancer models by reprogramming the nuclear–cytoplasmic trafficking of DNA-repair and stress-response factors (see Section 3.2).

Beyond these classical import receptors, recent evidence implicates Importin-7 (IPO7) as another actionable determinant of drug resistance [112]. In nasopharyngeal carcinoma, circIPO7 facilitates the nuclear import of Y-box-binding protein 1 (YBX1) via IPO7, promoting fibroblast growth factor receptor 1 (FGFR1)- and neurotrophic receptor tyrosine kinase 1 (NTRK1)-driven transcriptional programs and cisplatin resistance. Genetic silencing of circIPO7 or pharmacologic blockade of IPO7 prevented YBX1 nuclear accumulation and restored chemosensitivity in both cell lines and xenografts.

Direct inhibition of nuclear export by XPO1/CRM1 blocking is the most clinically advanced strategy to target NCT. Selinexor, first-in-class SINE inhibitor, recovers nuclear retention of tumor suppressive proteins such as p53, retinoblastoma protein (RB), and FOXO, thereby resensitizing refractory cancer cells. For example, in multiple myeloma, Wang et al. showed that Lipin1 is accumulated into nucleus upon XPO1 inhibition, suppressing sterol regulatory element-binding protein (SREBP)-dependent lipogenesis and resensitizing MM cells to selinexor [113]. As another example, in relapsed/refractory T-lymphoblastic leukemia model, Meng et al. showed that XPO1 inhibitor selinexor plus decitabine yields their synergistic increase of cytotoxicity by tumor suppressive protein nuclear retention [114]. More recently, selinexor has also been shown to overcome radioresistance in esophageal squamous-cell carcinoma by blocking XPO1-dependent export of p53, leading to augmented DNA-damage–induced apoptosis and radiosensitization [115].

Beyond single-receptor targeting, novel small molecules are emerging that concurrently inhibit multiple importins to reprogram nuclear trafficking networks. A recent study showed that the sphingosine analog SH-BC-893 directly binds Importin-β1, transportin 1 (TNPO1), importin 5 (IPO5), and IPO7, thereby preventing nuclear import of YAP, MYC, and NF-κB and restoring therapeutic sensitivity in resistant models [116].

These works hint at the following generalizable principle: in selected contexts, inhibiting dysregulated import or export receptors can restore nuclear residence of tumor suppressors or pro-apoptotic proteins and thereby resensitize cancer cells to therapy. Receptor targeting is therapeutically attractive because small-molecule inhibitors are available, but clinical translation requires tumor-specific cargo dependency, toxicity management, and biomarker-guided patient selection.

### 4.3 Targeting Signaling Pathways and NCT Crosstalk

One critical therapeutic aspect entails crosstalk between oncogenic signal transduction and NCT such that signal-driven protein localization changes lead to resistance [117].

PI3K–AKT–FOXO pathway: A recent example comes from AML, where phosphoproteomic and spatial-proteomic analyses of primary patient samples identified activated AKT–FOXO3 signaling as a resistance mechanism to selinexor. Mechanistically, AKT inhibition with MK-2206 promoted FOXO3 nuclear translocation and cooperated with selinexor to suppress AML cell proliferation, indicating that restoring the nuclear localization of a signaling-regulated tumor suppressor can enhance the response to nuclear export blockade [118]. Similarly, in multidrug-resistant NSCLC, targeting the PI3K–AKT–FOXO pathway with the dual PI3K/mechanistic target of rapamycin (mTOR) inhibitor DHW-221 reversed Akt-dependent FOXO3a cytoplasmic mislocalization, promoted FOXO3a nuclear translocation, and thereby contributed to Taxol resistance reversal in A549/Taxol cells and xenograft models [119].

NF-κB pathway: In uterine leiomyosarcoma models, selinexor caused nuclear retention of nuclear retention of inhibitor of NF-κB alpha (IκB-α), reduced NF-κB nuclear translocation, and sensitized tumors to eribulin chemotherapy [120]. Moreover, in triple-negative breast cancer (TNBC), Paulson et al. showed treatment leads to increased nuclear retention of NF-κB inhibitor alpha (NFKBIA), leading to NF-κB signaling suppression (such as G2/M checkpoint genes and E2F/Myc targets down-regulation), and NFKBIA knock-down decreases substantially selinexor sensitivity [121].

p53–DNA repair pathway: Selinexor increased radiosensitivity in esophageal carcinoma through accumulation of p53 in the nucleus and inhibition of DNA repair protein function, thus expanding radiation response [115].

In combination, these results underscore NCT’s intersection with principal cascades—PI3K–AKT–FOXO, NF-κB, and p53–DNA repair. These examples support these axes as rational combination contexts for NCT-directed therapy, but they should still be framed mainly as preclinical or *ex vivo* evidence rather than as clinically mature strategies comparable to approved XPO1-directed regimens.

### 4.4 Indirect Targeting through PTMs

PTMs usually determine concealment/exposure of NLS or NES and therefore alter subcellular localization of critical proteins and determine drug responses [1]. As discussed in preceding sections, several phosphorylation-driven examples have demonstrated that modulation of PTM-dependent nuclear import or export can effectively reverse resistance. For instance, in colorectal cancer, platinum-induced p38 MAPK activation phosphorylates GCLM at Thr17 to promote importin-α5-mediated nuclear import and oxaliplatin tolerance, whereas depletion of GCLM re-sensitizes resistant cells to chemotherapy [68] (see Section 3.3.2). Beyond phosphorylation, cisplatin-induced 6-phosphofructo-2-kinase/fructose-2,6-bisphosphatase 3 (PFKFB3) K472 acetylation disrupts an NLS-associated importin-α5 interaction, prevents PFKFB3 nuclear translocation, and promotes cytoplasmic glycolytic activity that protects cancer cells from cisplatin-induced apoptosis; enforced nuclear localization abolishes this protection, supporting a causal link between PTM-regulated localization and drug response [122]. PTMs as upstream molecular switches that fine-tune nucleocytoplasmic transport and offer actionable leverage points for overcoming resistance-associated phenotypes.

Among PTM-directed strategies, methylation-regulating enzymes such as PRMTs and SET-domain proteins represent potential therapeutic targets [123]. Some of the PRMT-specific small-molecule inhibitors are in preclinical or early clinical trials [124], and restoring drug sensitivity by normalizing aberrant protein localization is an exciting possibility [125]. However, daunting challenges remain delineating tumor-type specificity of methylation-regulated NCT events, untangling their interactions with other post-translational modifications, and identification of predictive biomarkers to classify patients likely to benefit [126]. Closure of these gaps will be important to achieving methylation–NCT biology’s promise of actionable means of bypassing resistance.

### 4.5 Indirect Targeting through Microenvironment

Tumor resistance can also arise from adaptive changes in the microenvironment, such as hypoxia, acidosis, and lactate accumulation [127]. Targeting these stress conditions offers an indirect yet effective approach to restore drug sensitivity.

Therapeutic targeting of microenvironment-induced resistance remains best supported for specific stress pathways rather than for NCT reversal as a general class. In esophagus and colorectal cancer, hypoxia-inducible factor 1 alpha (HIF-1α) inhibition by small molecule agents, like PX-478, increased substantially the efficacy of oxaliplatin or cisplatin, and it reduced tumor burden *in vivo* [128,129,130].

Acidic tumor microenvironments can repress mTORC1 inhibitor activity; oral alkalinisation by sodium bicarbonate elevated intratumoral pH and increased therapeutic efficiency in mouse models [131]. Hypoxia induces HIF-1α-dependent carbonic anhydrase IX (CAIX) expression, which supports extracellular acidosis and helps tumor cells tolerate microenvironmental stress [132]. In gastric cancer, CAIX expression was higher in patients who failed to respond to perioperative chemotherapy, and drug-resistant gastric cancer cells showed increased CAIX expression; inhibition of CAIX with SLC-0111 re-sensitized resistant cells to 5-fluorouracil, taxane-derived agents, and platinum-based drugs [133]. In immunotherapy models, CAIX inhibition also enhanced immune-checkpoint blockade by reducing acidic TME-associated immune suppression and improving antitumor T helper 1 (Th1) responses [134].

Additionally, lactate metabolism represents another therapeutic microenvironmental axis: Pharmacological inhibition of the lactate transporter lactate transporter monocarboxylate transporter 1 (MCT1) by AZD3965 suppressed tumor growth in diffuse large B-cell lymphoma and Burkitt lymphoma and showed synergy in combination with doxorubicin or rituximab [135].

Collectively, these studies demonstrate that normalizing hypoxia, acidosis, or lactate accumulation can re-sensitize refractory tumors, although direct *in vivo* evidence linking microenvironment-induced nuclear–cytoplasmic mislocalization to resistance reversal remains to be established.

### 4.6 Genetic Correction of Localization Signals

The process of directly editing nuclear localization signals (NLS) or nuclear export signals (NES) to reprogram protein subcellular localization is recent, but systematic therapeutic editing or correction of these motifs remains an emerging proof-of-concept strategy. As an illustration, one recent paper found and edited a NES motif in EGFR (aa 736–749); mutation at this position impaired nuclear export and resulted in elevated nuclear accumulation of EGFR, which corresponded to heterogeneous therapeutic responses to tyrosine kinase inhibitor [96]. In a parallel manner, functional studies of breakpoint cluster region-Abelson (BCR-ABL) demonstrated that mutation or deletions reestablishing function of latent NLS elements elevated partial nuclear localization of an oncoprotein [136]. Moreover, motif-based and deep-learning-enabled NLS prediction [137], curated NES-containing CRM1 cargo databases [138], activity-based NES prediction tools [139], and proteome-wide screens for cancer mutations that disrupt NES function [140] have provided a foundation for identifying localization-signal-regulated cargoes and prioritizing candidate NLS/NES alterations for functional validation.

These studies provide proof-of-concept that genetic editing of localization motifs can reprogram nuclear–cytoplasmic distribution of key proteins, yet direct evidence showing restoration of drug sensitivity after NLS/NES correction remains lacking. Future integration of CRISPR or prime-editing models with localization and drug-response assays may clarify whether such targeted correction can overcome chemoresistance.

**Table 4:** Representative strategies for targeting NCT-associated resistance phenotypes.

Therapeutic Class	Target/Intervention	NCT-Based Reversal Mechanism	Cancer Context	Resensitization Outcome	Ref.
NPC structure/permeability modulation	Nuclear envelope mechanics; lamin–NPC coupling	Mechanical modulation of the nuclear envelope alters NPC gating and enhances nuclear accumulation of doxorubicin	Breast cancer models	Restores doxorubicin efficacy in previously resistant cells	[33]
Direct targeting of import receptors	KPNB1/importin-β1 inhibition	Reprograms nuclear–cytoplasmic trafficking by restoring p53 stabilization/activation and reducing NF-κB nuclear translocation	Cervical cancer	Restores cisplatin sensitivity	[55]
	IPO4 inhibition	Prevents nuclear import of CEBPD, thereby attenuating PRKDC (DNA-PKcs)-mediated DNA repair	Cervical cancer	Restores cisplatin sensitivity	[57]
	circIPO7 silencing or IPO7 blockade	Blocks IPO7-dependent nuclear import of YBX1 and suppresses FGFR1/NTRK1-driven transcriptional programs	Nasopharyngeal carcinoma	Restores cisplatin sensitivity in cells and xenografts	[112]
Direct targeting of export receptors	XPO1 inhibition (selinexor)	Promotes nuclear accumulation of Lipin1 and suppresses SREBP-dependent lipogenesis	Multiple myeloma	Re-sensitizes myeloma cells to selinexor	[113]
Direct targeting of export receptors/combination strategy	Selinexor + decitabine	Enhances nuclear retention of tumor-suppressive proteins	Relapsed/refractory T-lymphoblastic leukemia	Synergistically increases cytotoxicity	[114]
	Selinexor-mediated p53 nuclear retention	Blocks XPO1-dependent p53 export, increases nuclear p53, and inhibits DNA repair-related resistance programs	Esophageal squamous-cell carcinoma	Reverses radioresistance and enhances radiosensitivity	[115]
Multi-importin blockade	SH-BC-893	Simultaneously inhibits KPNB1, TNPO1, IPO5, and IPO7, preventing nuclear import of YAP, MYC, and NF-κB	Resistant tumor models	Restores therapeutic sensitivity	[116]
NCT–signaling crosstalk targeting	Selinexor + AKT inhibition (MK-2206)	Promotes FOXO3 nuclear translocation and cooperates with nuclear export blockade	AML primary samples/models	Suppresses selinexor-resistant AML cell proliferation in *ex vivo* and model systems	[118]
	DHW-221, dual PI3K/mTOR inhibition	Suppresses Akt signaling and promotes FOXO3a nuclear translocation	Taxol-resistant NSCLC cells/xenografts	Overcomes multidrug resistance and induces apoptosis	[119]
	Selinexor + eribulin	Causes nuclear retention of IκB-α and reduces NF-κB nuclear signaling	Uterine leiomyosarcoma	Sensitizes tumors to chemotherapy	[120]
	Selinexor	Increases nuclear retention of NFKBIA and suppresses NF-κB-associated transcriptional programs	Triple-negative breast cancer	Enhances selinexor sensitivity; NFKBIA loss reduces response	[121]
Indirect targeting through PTMs	GCLM depletion/targeting p38–GCLM–importin-α5 axis	Disrupts phosphorylation-driven importin-α5-mediated nuclear import of GCLM	Colorectal cancer	Re-sensitizes oxaliplatin-resistant cells	[68]
	Restoring PFKFB3 nuclear localization/targeting K472 acetylation	Counteracts acetylation-disrupted importin-α5 interaction and cytoplasmic glycolytic protection	Cisplatin-treated cancer models	Enforced nuclear localization abolishes cytoplasmic PFKFB3-mediated protection	[122]
	PRMT/SET-domain enzyme inhibition	Potentially normalizes methylation-regulated NCT events	Multiple cancers	Investigational; localization-based resistance reversal remains to be validated	[123,124,125,126]
Indirect targeting through microenvironment	HIF-1α inhibition (e.g., PX-478)	Attenuates hypoxia-driven resistance programs associated with maladaptive nuclear signaling	Esophageal and colorectal cancer	Increases cisplatin/oxaliplatin efficacy and reduces tumor burden *in vivo*	[128,129,130]
	CAIX inhibition (SLC-0111)	Normalizes acidosis-associated resistance states downstream of hypoxia	Gastric cancer and immunotherapy-responsive tumor models	Re-sensitizes resistant cells to 5-FU, taxane-derived agents, and platinum-based drugs; improves checkpoint blockade in models	[131,132,133,134]
	MCT1 inhibition (AZD3965)	Targets lactate-dependent metabolic adaptation linked to resistant tumor states	DLBCL and Burkitt lymphoma	Suppresses tumor growth and synergizes with doxorubicin or rituximab	[135]
Genetic correction of localization determinants (proof-of-concept)	Editing of NLS/NES motifs	Reprograms nuclear–cytoplasmic distribution of key proteins by correcting localization determinants	EGFR, BCR-ABL	Proof-of-concept only; direct restoration of drug sensitivity remains to be demonstrated	[96,136]

Abbreviations: NCT, nucleocytoplasmic transport; NPC, nuclear pore complex; KPNB1, karyopherin subunit beta 1; IPO4, importin-4; IPO7, importin-7; YBX1, Y-box-binding protein 1; FGFR1, fibroblast growth factor receptor 1; NTRK1, neurotrophic receptor tyrosine kinase 1; XPO1/CRM1, exportin 1; RB, retinoblastoma protein; FOXO, forkhead box O; SREBP, sterol regulatory element-binding protein; PI3Kδ, phosphoinositide 3-kinase delta; IκB-α/NFKBIA, NF-κB inhibitor alpha; TNPO1, transportin 1; MYC, MYC proto-oncogene; NF-κB, nuclear factor kappa B; GCLM, glutamate-cysteine ligase modifier subunit; HIF-1α, hypoxia-inducible factor 1 alpha; CAIX, carbonic anhydrase IX; HNSCC, head and neck squamous cell carcinoma; MCT1, monocarboxylate transporter 1; DLBCL, diffuse large B-cell lymphoma; DNA-PKcs/PRKDC, DNA-dependent protein kinase catalytic subunit; NLS, nuclear localization signal; NES, nuclear export signal; PFKFB3, 6-phosphofructo-2-kinase/fructose-2,6-bisphosphatase 3; PRMT, protein arginine methyltransferase; mTOR, mechanistic target of rapamycin; 5-FU, 5-fluorouracil.

## 5 Clinical Trials

Among NCT-targeted strategies, XPO1/CRM1 inhibition has the most mature clinical evidence. Selinexor was the first-in-class selective inhibitor of nuclear export (SINE) to receive regulatory approval, and its strongest activity has been observed in hematologic malignancies such as relapsed/refractory multiple myeloma (MM) and diffuse large B-cell lymphoma (DLBCL) [141]. In the STORM trial (NCT02336815) [22], selinexor plus dexamethasone produced an objective response rate (ORR) of approximately 26% in triple-class refractory MM was achieved through combining selinexor dexamethasone, otherwise poor-prognosis disease. The BOSTON trial (NCT03110562) also demonstrated that weekly selinexor combined with bortezomib and dexamethasone significantly improved progression-free survival (13.9 vs. 9.5 months), supporting regulatory approval in MM [23]. More recent MM studies have extended this combination logic to anti-CD38 antibody- and carfilzomib-containing regimens: the phase II GEM-SELIBORDARA study (NCT03589222) evaluated selinexor with daratumumab, bortezomib, and dexamethasone, and a phase I study (NCT02199665) supported the feasibility of selinexor with weekly carfilzomib and dexamethasone in relapsed/refractory MM [110,142]. The SADAL trial (NCT02227251) in DLBCL also demonstrated durable responses to single-agent selinexor in selected patients [143]. Selinexor is being evaluated with ruxolitinib in the phase III SENTRY trial [144] (NCT04562389) for myelofibrosis, and selinexor with venetoclax (NCT03955783) has also shown clinical activity in relapsed/refractory acute myeloid leukemia [145], highlighting the ongoing expansion of XPO1-directed therapy beyond multiple myeloma and lymphoma.

In comparison, solid tumor outcomes have been modest. In the first-in-human advanced solid tumor study NCT01896505 [146], selinexor produced mostly stable disease with occasional partial responses, while anorexia and fatigue were common. In pediatric/adolescent recurrent or refractory solid and CNS tumors (NCT02323880) [147], objective responses were rare, although dosing parameters were established. In recurrent glioblastoma (NCT01986348) [148], selinexor monotherapy produced infrequent objective responses, although tumor reduction and disease stabilization were observed in some patients. More recent solid-tumor studies reinforce this mixed picture: the GEMS-001 phase II study (NCT02069730) [149] in recurrent or metastatic salivary gland tumors reported limited single-agent antitumor activity despite tumor reduction in some patients; similarly, in a phase Ib study of selinexor combined with standard chemotherapy in advanced or metastatic solid tumors (NCT02419495) [150], disease control was observed in some patients, but objective activity remained limited. By contrast, a phase I/II study (NCT03095612) [151] of selinexor plus docetaxel in previously treated KRAS-mutant NSCLC suggested manageable safety and activity in selected molecular contexts, with outcomes differing by TP53 status. These findings support the view that rational combinations and biomarker-guided selection may be required outside hematologic contexts. These differences may reflect tumor-intrinsic cargo dependencies, microenvironmental barriers, compensatory signaling, and drug tolerability, although the relative contribution of each factor remains context dependent [31].

The clinical use of SINE therapy is also constrained by toxicity and resistance [110,147]. Common clinically important adverse events include thrombocytopenia, fatigue, anorexia, nausea, and other toxicities that often require dose modification or supportive care [110,142]. Resistance to XPO1 inhibition also requires explicit consideration. Mutation of the XPO1 Cys528 drug-binding residue has been reported as a mechanism of resistance to selective inhibitors of nuclear export, providing a direct example of intrinsic or acquired resistance to NCT-targeted therapy [152,153]. Functionally, resistance can also arise from compensatory survival signaling, including NF-κB activity, which helps explain why combinations with proteasome inhibitors, DNA-damaging agents, PI3K-pathway inhibitors, or other context-specific therapies are being explored rather than relying on export blockade alone [118].

In response to this, new agents remain actively in development. Eltanexor, a second-generation selective inhibitor of nuclear export (SINE), is designed to retain antitumor activity while improving tolerability relative to selinexor, particularly by reducing central nervous system toxicity. Clinical activity has been greatest in hematologic malignancies. In NCT02649790 [154], eltanexor monotherapy in higher-risk myelodysplastic syndrome (MDS) refractory to hypomethylating agents yielded an overall response rate (ORR) of approximately 53%, marrow complete remissions (mCR) in nearly half of patients, and a median overall survival (OS) of approximately 9.9 months. A phase I study in refractory/relapsed multiple myeloma confirmed safety and disease stabilization in a subset of patients [155]. Combination strategies are also under active investigation: NCT06399640 [156] evaluates eltanexor in combination with venetoclax in refractory/relapsed MDS. Notably, although NCT02649790 [154] included expansion arms in solid tumors (e.g., metastatic colorectal cancer and mCRPC), published data to date have not manifested strong efficacy signals, suggesting a translational pattern like selinexor: stronger activity in hematologic malignancies but limited evidence in solid tumors.

KPT-9274, also known as padnarsertib, is a potentially first-in-class, orally bioavailable dual inhibitor of PAK4 (p21-activated kinase 4) and NAMPT (nicotinamide phosphoribosyltransferase) [157]. PAK4 contributes to cytoskeletal dynamics, nuclear signal transduction, and therapy-resistant phenotypes, whereas NAMPT is the rate-limiting enzyme of the nicotinamide adenine dinucleotide (NAD^+^) salvage pathway that supports tumor metabolism. As an inhibitor of both axes simultaneously, KPT-9274 disrupts both oncogenic signal transduction and tumor cell support of metabolism [157,158]. Preclinical studies supported its clinical translation by showing that KPT-9274 suppresses PAK4/NAMPT-dependent oncogenic signaling, metabolic support, drug resistance, and stemness-associated phenotypes in pancreatic ductal adenocarcinoma models, and can sensitize pancreatic neuroendocrine tumor models to everolimus through effects on rapamycin-insensitive companion of mTOR/mammalian target of rapamycin complex 2 (RICTOR/mTORC2) and β-catenin signaling [159,160]. Recently, the first-in-human phase I study KCP-9274-901 (NCT02702492) [157] evaluated KPT-9274 alone, with niacin, or with nivolumab in patients with advanced solid malignancies. A total of 60 patients were enrolled, including 50 patients in the dose-escalation parts and 10 patients in the nivolumab-combination part. The maximum tolerated dose was not reached in the single-agent/niacin dose-escalation cohorts, whereas the combination cohort established a tolerated dose of KPT-9274 with nivolumab. The most frequently reported treatment-emergent adverse events included anemia, arthralgia, and fatigue. Importantly, enrollment was halted prematurely because no observable clinical efficacy was detected across the study parts, indicating that KPT-9274 remains an early-stage, investigational strategy rather than a clinically validated NCT-directed therapy [157]. Together, KPT-9274 illustrates an indirect approach to modulating signaling and metabolic programs across the nucleus and cytoplasm and remains less clinically mature than direct XPO1 inhibition. The complete collection of NCT trials, including experimental combination trials, appears in Table 5.

In aggregate, clinical data support a staged interpretation of NCT-directed therapy: XPO1 inhibition is clinically validated in selected hematologic malignancies; XPO1-based combinations in solid tumors remain investigational; second-generation SINEs and indirect NCT modulators such as KPT-9274 are still in clinical testing; and most NPC-, importin-, PTM-, microenvironment-, or localization-signal-directed interventions remain preclinical or proof-of-concept. Future trials should therefore incorporate biomarker-guided patient selection, cargo-localization readouts, and separate reporting for hematologic versus solid tumor settings.

**Table 5:** Registered Clinical Trials of NCT-targeted therapies.

NCT No.	Cancer Type/Population	Phase/Design	Intervention	Status/Notes	Tumor Setting	Development/Evidence Stage	Ref.
NCT02336815 (STORM)	Triple-class refractory MM	Ph2	Selinexor + dexamethasone	Completed; ORR ~26%, approval-supporting MM evidence	Hematologic malignancy	Clinically validated; approval-supporting phase II evidence	[22]
NCT03110562 (BOSTON)	RRMM (1–3 prior lines)	Ph3, RCT	Selinexor + bortezomib + dexamethasone	Completed; PFS benefit, approval-supporting MM evidence	Hematologic malignancy	Clinically validated; phase III evidence	[23]
NCT03589222 (GEM-SELIBORDARA)	R/R MM	Ph2	Selinexor + daratumumab + bortezomib + dexamethasone	Active, not recruiting; phase II MM combination evidence	Hematologic malignancy	Investigational phase II combination	[110]
NCT02199665	R/R MM	Ph1	Selinexor + carfilzomib + dexamethasone	Completed; feasibility and dose-finding for carfilzomib-containing regimen	Hematologic malignancy	Early clinical combination testing	[142]
NCT02227251 (SADAL)	R/R DLBCL	Ph2, single-arm	Selinexor monotherapy	Active, not recruiting; ORR ~28% in selected patients	Hematologic malignancy	Clinically validated; approval-supporting phase II evidence	[143]
NCT04562389 (SENTRY)	JAK inhibitor treatment-naive myelofibrosis	Ph3 study design	Selinexor + ruxolitinib	Active, not recruiting; study-design evidence, not mature efficacy evidence	Hematologic malignancy	Ongoing phase III investigational combination	[144]
NCT03955783	Relapsed/refractory AML	Ph1b	Selinexor + venetoclax	Completed; clinical activity reported in R/R AML	Hematologic malignancy	Investigational early clinical combination	[145]
NCT01896505	Advanced refractory bone or soft-tissue sarcoma	Ph1/phase IB dose expansion	Selinexor monotherapy	Completed; safety/PK and limited activity in sarcoma	Solid tumor	Early clinical; completed phase I/IB	[146]
NCT02323880 (ADVL1414)	Pediatric/adolescent recurrent or refractory solid and CNS tumors	Ph1	Selinexor monotherapy	Completed; MTD defined, objective responses uncommon	Solid/CNS tumor	Early clinical dose-finding	[147]
NCT01986348	Recurrent glioblastoma	Ph2	Selinexor monotherapy	Completed; infrequent objective responses with tumor reduction/stabilization in some patients	CNS tumor	Exploratory phase II solid/CNS tumor evidence	[148]
NCT02069730 (GEMS-001)	Recurrent or metastatic salivary gland cancers	Molecularly assigned basket study; no phase listed	Selinexor-containing molecularly guided therapy	Completed; limited single-agent antitumor activity	Solid tumor	Exploratory solid-tumor evidence	[149]
NCT02419495	Advanced or metastatic solid tumors	Ph1	Selinexor + standard chemotherapy or immunotherapy agents	Terminated; combination safety and disease-control focus	Solid tumor	Early clinical combination testing	[150]
NCT03095612	Previously treated KRAS-mutant NSCLC	Ph1/2	Selinexor + docetaxel	Terminated; manageable safety and selected activity, with TP53-dependent signal	Solid tumor	Investigational phase I/II combination	[151]
NCT02649790	R/R cancer indications including HR-MDS, RRMM, AML, mCRC, and mCRPC	Ph1/2	Eltanexor monotherapy	Completed; strongest published activity in HMA-refractory MDS; limited solid-tumor signals	Mixed; mainly hematologic signal	Investigational second-generation SINE evidence	[154]
NCT06399640	R/R MDS and AML	Ph1	Eltanexor + venetoclax	Recruiting; rational combination strategy under active evaluation	Hematologic malignancy	Ongoing early clinical combination trial	[156]
NCT02702492 (PANAMA)	Advanced solid malignancies or NHL	Ph1	KPT-9274/padnarsertib	Terminated; tolerable dosing, disease stabilization in some patients, limited objective efficacy	Mixed; mainly solid-tumor enrollment	Early clinical; indirect NCT-modulating strategy	[157]

Abbreviations: NCT, nucleocytoplasmic transport; MM, multiple myeloma; DLBCL, diffuse large B-cell lymphoma; CNS, central nervous system; MDS, myelodysplastic syndromes; NHL, non-Hodgkin lymphoma; ORR, objective response rate; PFS, progression-free survival; mCR, marrow complete remission; OS, overall survival; XPO1, exportin 1; PAK4, p21-activated kinase 4; NAMPT, nicotinamide phosphoribosyltransferase; R/R, relapsed/refractory; HR-MDS, higher-risk myelodysplastic syndrome; mCRC, metastatic colorectal cancer; mCRPC, metastatic castration-resistant prostate cancer; SVd, selinexor, bortezomib and dexamethasone; PK, pharmacokinetics.

## 6 Challenges and Future Directions

NCT-targeted therapies have shown the clearest benefit in selected hematologic malignancies and in refractory settings. However, several barriers must be addressed before NCT modulation can be broadly applied as an oncology strategy. The next phase of the field should move from pathway-wide inhibition toward cargo-specific biomarkers, rational combinations, and tumor-context-aware dosing strategies.

### 6.1 Tumor Selectivity of NCT Inhibition

XPO1 and nuclear pore proteins are also expressed in normal cells; therefore, systemic inhibition can cause adverse effects in the nervous system and in bone marrow. Future therapeutic strategies should preferentially focus on those tumors whose survival depends heavily on certain exported proteins, such as NF-κB regulators or DNA-repair proteins, thereby widening the therapeutic window between malignant and normal tissues.

### 6.2 Potential Resistance Mechanisms

Emerging evidence suggests tumors may adapt through mutation in XPO1, activation of compensatory transport pathways, or alterations in cargo proteins. These mechanisms support the need for next-generation inhibitors, intermittent or toxicity-adapted dosing, and rational combination strategies targeting the dominant cargo dependencies within specific tumor contexts.

### 6.3 NCT Cargo Profiles as Biomarkers

Different tumors rely on different exported cargo sets; some depend on DNA-repair factors, while others require metabolic enzymes or transcriptional regulators. Proteomics or single-cell level visualization of such cargo profiles can help predict patient response and track treatment impacts.

### 6.4 Synergy with Immunotherapy

NCT inhibitors may promote the nuclear retention of tumor suppressors and attenuate immunosuppressive signaling, thereby reshaping the tumor microenvironment. These effects provide a rationale for combining NCT-targeted therapies with immune checkpoint inhibitors or cell-based immunotherapies such as CAR-T cells, but these strategies require careful validation because immunological consequences may differ substantially between hematologic malignancies and solid tumors.

In summary, improving tumor selectivity, preventing resistance, identifying predictive biomarkers, and defining rational immune combinations represent key future directions for NCT inhibitor research. Progress in these areas will determine whether NCT-directed drugs remain salvage therapies or become integrated components of targeted cancer treatment.

## Data Availability

Data sharing not applicable to this article as no new datasets were generated in this study. All data analyzed and summarized from published literature are included in this published article (and its supplementary information files).

## Supplementary Materials

The supplementary material is available online at https://www.techscience.com/doi/10.32604/or.2026.083902/s1. Table S1: NCT–signaling crosstalk driving therapy resistance. Table S2: Representative examples of NLS/NES mutations that alter protein localization and modulate anticancer drug responses. Table S3: Representative studies linking TME pressures to NCT-dependent therapeutic resistance.

## Abbreviations

5-FU	5-fluorouracil
53BP1	p53-binding protein 1
ACLY	ATP-citrate lyase
ADVL1414	Children’s Oncology Group phase I consortium trial identifier
ALL	acute lymphoblastic leukemia
AML	acute myeloid leukemia
ANOVA	analysis of variance
AR	androgen receptor
AR-V7	androgen receptor splice variant 7
BCR-ABL	breakpoint cluster region-Abelson
BRCA1	breast cancer susceptibility gene 1
CAIX	carbonic anhydrase IX
CCNU	lomustine
CDK2	cyclin-dependent kinase 2
CDK4/6	Cyclin-dependent kinases 4 and 6
CEBPD	CCAAT/enhancer-binding protein delta
CLL	chronic lymphocytic leukemia
CNS	central nervous system
CRM1	chromosome region maintenance 1, also known as exportin 1
CYP2C9	cytochrome P450 family 2 subfamily C member 9
DD-LPS	dedifferentiated liposarcoma
DLBCL	diffuse large B-cell lymphoma
DNA-PKcs	DNA-dependent protein kinase catalytic subunit
DSB	DNA double-strand break
E2F1	E2F transcription factor 1
ECM	extracellular matrix
EGFR	epidermal growth factor receptor
EMT	epithelial-mesenchymal transition
ERK	extracellular signal-regulated kinase
FDA	Food and Drug Administration
FG	phenylalanine-glycine-rich
FGFR1	fibroblast growth factor receptor 1
FOXO	forkhead box O
FZD7	frizzled class receptor 7
GBM	glioblastoma
GCLM	glutamate-cysteine ligase modifier subunit
GPR81/HCAR1	hydroxycarboxylic acid receptor 1
GSK3β	glycogen synthase kinase 3 beta
HIF-1α	hypoxia-inducible factor 1 alpha
HMA	hypomethylating agent
HNSCC	head and neck squamous cell carcinoma
HR	hazard ratio
HR-MDS	higher-risk myelodysplastic syndrome
IκB-α	NF-κB inhibitor alpha
IPO4	importin-4
IPO7	importin-7
JAK1	Janus kinase 1
Keap1	Kelch-like ECH-associated protein 1
KPNA1	karyopherin subunit alpha 1
KPNB1	karyopherin subunit beta 1
KRAS	Kirsten rat sarcoma viral oncogene homolog
LLPS	liquid-liquid phase separation
MAPK	mitogen-activated protein kinase
MCF7-ADR	Michigan Cancer Foundation-7/adriamycin-resistant
mCR	marrow complete remission
mCRC	metastatic colorectal cancer
mCRPC	metastatic castration-resistant prostate cancer
MCT1	monocarboxylate transporter 1
MDS	myelodysplastic syndromes
MITF	microphthalmia-associated transcription factor
MM	multiple myeloma
MTD	maximum tolerated dose
mTOR	mechanistic target of rapamycin
MYC	MYC proto-oncogene
NAD+	nicotinamide adenine dinucleotide
NAMPT	nicotinamide phosphoribosyltransferase
NCT	nucleocytoplasmic transport
NEK6	NIMA-related kinase 6
NES	nuclear export signal
NF-κB	nuclear factor kappa B
NFKBIA	NF-κB inhibitor alpha
NHEJ	non-homologous end joining
NHL	non-Hodgkin lymphoma
NKRF	NF-κB-repressing factor
NLS	nuclear localization signal
NPC	nuclear pore complex
NRF2	nuclear factor erythroid 2-related factor 2
NSCLC	non-small cell lung cancer
NUP153	nucleoporin 153
NUP358	nucleoporin 358
NUP98	nucleoporin 98
OGT	O-GlcNAc transferase
ORR	objective response rate
OS	overall survival
P-gp	P-glycoprotein
PAK2	p21-activated kinase 2
PAK4	p21-activated kinase 4
PARP	poly(ADP-ribose) polymerase
PBK	PDZ-binding kinase
PDAC	pancreatic ductal adenocarcinoma
PFKFB3	6-phosphofructo-2-kinase/fructose-2,6-bisphosphatase 3
PFS	progression-free survival
PI3Kα	phosphoinositide 3-kinase alpha
PI3Kδ	phosphoinositide 3-kinase delta
PIK3R1	phosphoinositide-3-kinase regulatory subunit 1
PK	pharmacokinetics
PR	partial response
PRKDC	protein-coding gene symbol for DNA-dependent protein kinase catalytic subunit
PRMT	protein arginine methyltransferase
PTM	post-translational modification
PUMA	p53 upregulated modulator of apoptosis
R/R	relapsed/refractory
RB	retinoblastoma protein
RCT	randomized controlled trial
RFNG	RFNG O-fucosylpeptide 3-beta-N-acetylglucosaminyltransferase
RICTOR	rapamycin-insensitive companion of mTOR
RPN2	ribophorin II
RRMM	relapsed/refractory multiple myeloma
RT	radiotherapy
SD	standard deviation
SEM	standard error of the mean
SGK3	serum/glucocorticoid-regulated kinase 3
SINE	selective inhibitor of nuclear export
SN38	7-ethyl-10-hydroxycamptothecin
SREBP	sterol regulatory element-binding protein
STAT3	signal transducer and activator of transcription 3
SUMO	small ubiquitin-like modifier
SV40	simian virus 40
SVd	selinexor, bortezomib and dexamethasone
T-ALL	T-cell acute lymphoblastic leukemia
TAZ	transcriptional coactivator with PDZ-binding motif
TLR3	toll-like receptor 3
TME	tumor microenvironment
TMZ	temozolomide
TNBC	triple-negative breast cancer
TNPO1	transportin 1
TOP1	topoisomerase I
TP53	tumor protein p53
TRIM25	tripartite motif-containing protein 25
TRIM32	tripartite motif-containing protein 32
TRIM37	tripartite motif-containing protein 37
USP22	ubiquitin-specific peptidase 22
Vd	bortezomib and dexamethasone
WNT	Wingless/INT-1
XIAP	X-linked inhibitor of apoptosis protein
XPO1	exportin 1
XRCC4	X-ray repair cross-complementing protein 4
YAP	Yes-associated protein
YBX1	Y-box-binding protein 1

## References

[1] Yang Y , Guo L , Chen L , Gong B , Jia D , Sun Q . Nuclear transport proteins: Structure, function, and disease relevance. Signal Transduct Target Ther. 2023; 8( 1): 425. doi:10.1038/s41392-023-01649-4. 37945593 PMC10636164

[2] Khakwani MMAK , Ji XY , Khattak S , Sun YC , Yao K , Zhang L . Targeting colorectal cancer at the level of nuclear pore complex. J Adv Res. 2025; 70: 423– 44. doi:10.1016/j.jare.2024.06.009. 38876192 PMC11976419

[3] Yan S , Zhan F , He Y , Zhu Y , Ma Z . p53 in colorectal cancer: From a master player to a privileged therapy target. J Transl Med. 2025; 23( 1): 684. doi:10.1186/s12967-025-06566-4. 40537809 PMC12178040

[4] Jiang J , Yang ES , Jiang G , Nowsheen S , Wang H , Wang T , et al. p53-dependent BRCA1 nuclear export controls cellular susceptibility to DNA damage. Cancer Res. 2011; 71( 16): 5546– 57. doi:10.1158/0008-5472.CAN-10-3423. 21742769 PMC12912278

[5] Guo X , Peng K , He Y , Xue L . Mechanistic regulation of FOXO transcription factors in the nucleus. Biochim Biophys Acta Rev Cancer. 2024; 1879( 2): 189083. doi:10.1016/j.bbcan.2024.189083. 38309444

[6] Louault K , Blavier L , Lee MH , Kennedy RJ , Fernandez GE , Pawel BR , et al. Nuclear factor-κB activation by transforming growth factor-β1 drives tumour microenvironment-mediated drug resistance in neuroblastoma. Br J Cancer. 2024; 131( 1): 90– 100. doi:10.1038/s41416-024-02686-8. 38806726 PMC11231159

[7] Zhou W , Wang S , Zhang Z , Li L , Zhu J , Lin H , et al. Restoration of Osimertinib sensitivity in lung cancer through BRD4 inhibitor-mediated depalmitoylation of mutant EGFR via APT1. npj Precis Oncol. 2025; 9( 1): 305. doi:10.1038/s41698-025-01048-8. 40877429 PMC12394438

[8] Balasubramanian SK , Azmi AS , Maciejewski J . Selective inhibition of nuclear export: A promising approach in the shifting treatment paradigms for hematological neoplasms. Leukemia. 2022; 36( 3): 601– 12. doi:10.1038/s41375-021-01483-z. 35091658 PMC8885406

[9] Zaitsava H , Gachowska M , Bartoszewska E , Kmiecik A , Kulbacka J . The potential of nuclear pore complexes in cancer therapy. Molecules. 2024; 29( 20): 4832. doi:10.3390/molecules29204832. 39459201 PMC11510365

[10] Zhang Y , Tan Y , Yuan J , Tang H , Zhang H , Tang Y , et al. circLIFR-007 reduces liver metastasis via promoting hnRNPA1 nuclear export and YAP phosphorylation in breast cancer. Cancer Lett. 2024; 592: 216907. doi:10.1016/j.canlet.2024.216907. 38685451

[11] Li Z , Zhu T , Wu Y , Yu Y , Zang Y , Yu L , et al. Functions and mechanisms of non-histone post-translational modifications in cancer progression. Cell Death Discov. 2025; 11( 1): 125. doi:10.1038/s41420-025-02410-2. 40164592 PMC11958777

[12] Chen Z , Han F , Du Y , Shi H , Zhou W . Hypoxic microenvironment in cancer: Molecular mechanisms and therapeutic interventions. Signal Transduct Target Ther. 2023; 8( 1): 70. doi:10.1038/s41392-023-01332-8. 36797231 PMC9935926

[13] Seymour L , Nuru N , Johnson KR , Gutierrez JMV , Njoku VT , Darie CC , et al. Roles of post-translational modifications of transcription factors involved in breast cancer hypoxia. Molecules. 2025; 30( 3): 645. doi:10.3390/molecules30030645. 39942749 PMC11820228

[14] Livneh I , Cohen-Kaplan V , Fabre B , Abramovitch I , Lulu C , Nataraj NB , et al. Regulation of nucleo-cytosolic 26S proteasome translocation by aromatic amino acids via mTOR is essential for cell survival under stress. Mol Cell. 2023; 83( 18): 3333– 46.e5. doi:10.1016/j.molcel.2023.08.016. 37738964

[15] Lin X , Chen W , Yang G , Zhang J , Wang H , Liu Z , et al. Viral infection induces inflammatory signals that coordinate YAP regulation of dysplastic cells in lung alveoli. J Clin Investig. 2024; 134( 19): e176828. doi:10.1172/JCI176828. 39352385 PMC11444164

[16] Li Y , Zhong Z , Xu C , Wu X , Li J , Tao W , et al. 3D micropattern force triggers YAP nuclear entry by transport across nuclear pores and modulates stem cells paracrine. Natl Sci Rev. 2023; 10( 8): nwad165. doi:10.1093/nsr/nwad165. 37457331 PMC10347367

[17] Miao C , Huang Y , Zhang C , Wang X , Wang B , Zhou X , et al. Post-translational modifications in drug resistance. Drug Resist Updat. 2025; 78: 101173. doi:10.1016/j.drup.2024.101173. 39612546

[18] Huang Q , Zhao R , Xu L , Hao X , Tao S . Treatment of multiple myeloma with selinexor: A review. Ther Adv Hematol. 2024; 15: 20406207231219442. doi:10.1177/20406207231219442. 38186637 PMC10771077

[19] Bruserud Ø , Selheim F , Hernandez-Valladares M , Reikvam H . XPO1/Exportin-1 in acute Myelogenous Leukemia; biology and therapeutic targeting. Biomolecules. 2025; 15( 2): 175. doi:10.3390/biom15020175. 40001478 PMC11852384

[20] Azmi AS , Uddin MH , Mohammad RM . The nuclear export protein XPO1—From biology to targeted therapy. Nat Rev Clin Oncol. 2021; 18( 3): 152– 69. doi:10.1038/s41571-020-00454-0. 33173198

[21] Li YQ , Fang Z , Zhang W , Rao GW , Zheng Q . Targeting XPO1 for fighting relapsed/refractory diseases: The research progress of XPO1 inhibitors. Bioorg Chem. 2025; 154: 108073. doi:10.1016/j.bioorg.2024.108073. 39708554

[22] Chari A , Vogl DT , Gavriatopoulou M , Nooka AK , Yee AJ , Huff CA , et al. Oral selinexor-dexamethasone for triple-class refractory multiple myeloma. N Engl J Med. 2019; 381( 8): 727– 38. doi:10.1056/NEJMoa1903455. 31433920

[23] Grosicki S , Simonova M , Spicka I , Pour L , Kriachok I , Gavriatopoulou M , et al. Once-per-week selinexor, bortezomib, and dexamethasone versus twice-per-week bortezomib and dexamethasone in patients with multiple myeloma (BOSTON): A randomised, open-label, phase 3 trial. Lancet. 2020; 396( 10262): 1563– 73. doi:10.1016/S0140-6736(20)32292-3. 33189178

[24] Kalderon D , Roberts BL , Richardson WD , Smith AE . A short amino acid sequence able to specify nuclear location. Cell. 1984; 39( 3 Pt 2): 499– 509. doi:10.1016/0092-8674(84)90457-4. 6096007

[25] Görlich D , Vogel F , Mills AD , Hartmann E , Laskey RA . Distinct functions for the two importin subunits in nuclear protein import. Nature. 1995; 377( 6546): 246– 8. doi:10.1038/377246a0. 7675110

[26] Mattaj IW , Englmeier L . Nucleocytoplasmic transport: The soluble phase. Annu Rev Biochem. 1998; 67: 265– 306. doi:10.1146/annurev.biochem.67.1.265. 9759490

[27] Fornerod M , Ohno M , Yoshida M , Mattaj IW . CRM1 is an export receptor for leucine-rich nuclear export signals. Cell. 1997; 90( 6): 1051– 60. doi:10.1016/S0092-8674(00)80371-2. 9323133

[28] Ishizawa J , Kojima K , Hail N , Tabe Y , Andreeff M . Expression, function, and targeting of the nuclear exporter chromosome region maintenance 1 (CRM1) protein. Pharmacol Ther. 2015; 153: 25– 35. doi:10.1016/j.pharmthera.2015.06.001. 26048327 PMC4526315

[29] Komlodi-Pasztor E , Trostel S , Sackett D , Poruchynsky M , Fojo T . Impaired p53 binding to importin: A novel mechanism of cytoplasmic sequestration identified in oxaliplatin-resistant cells. Oncogene. 2009; 28( 35): 3111– 20. doi:10.1038/onc.2009.166. 19581934 PMC2867326

[30] Yang ES , Nowsheen S , Rahman MA , Cook RS , Xia F . Targeting BRCA1 localization to augment breast tumor sensitivity to poly(ADP-ribose) polymerase inhibition. Cancer Res. 2012; 72( 21): 5547– 55. doi:10.1158/0008-5472.CAN-12-0934. 22962264 PMC3771348

[31] Lai C , Xu L , Dai S . The nuclear export protein exportin-1 in solid malignant tumours: From biology to clinical trials. Clin Transl Med. 2024; 14( 5): e1684. doi:10.1002/ctm2.1684. 38783482 PMC11116501

[32] Chen YF , Adams DJ . Therapeutic targeting of exportin-1 beyond nuclear export. Trends Pharmacol Sci. 2025; 46( 1): 20– 31. doi:10.1016/j.tips.2024.11.002. 39643565 PMC11711008

[33] Scott NR , Kang S , Parekh SH . Mechanosensitive nuclear uptake of chemotherapy. Sci Adv. 2024; 10( 51): eadr5947. doi:10.1126/sciadv.adr5947. 39693448 PMC11654694

[34] Rush C , Jiang Z , Tingey M , Feng F , Yang W . Unveiling the complexity: Assessing models describing the structure and function of the nuclear pore complex. Front Cell Dev Biol. 2023; 11: 1245939. doi:10.3389/fcell.2023.1245939. 37876551 PMC10591098

[35] Chen Y , Zhou G , Yu M . Conformational dynamics of the nuclear pore complex central channel. Biochem Soc Trans. 2025; 53( 1): 267– 79. doi:10.1042/BST20240507. 39927798

[36] Wu KP , Yan ZJ , Zhuang XX , Hua JL , Li MX , Huang K , et al. Dynamic structure and function of nuclear pore protein complex: Potential roles of lipid and lamins regulated nuclear membrane curvature. Int J Biol Macromol. 2025; 313: 144104. doi:10.1016/j.ijbiomac.2025.144104. 40350114

[37] Rodriguez-Bravo V , Pippa R , Song W-M , Carceles-Cordon M , Dominguez-Andres A , Fujiwara N , et al. Nuclear Pores promote lethal prostate cancer by increasing POM121-driven E2F1, MYC, and AR nuclear import. Cell. 2018; 174( 5): 1200– 15.e20. doi:10.1016/j.cell.2018.07.015. 30100187 PMC6150493

[38] Hazawa M , Lin DC , Kobayashi A , Jiang YY , Xu L , Dewi FRP , et al. ROCK-dependent phosphorylation of NUP62 regulates p63 nuclear transport and squamous cell carcinoma proliferation. EMBO Rep. 2017; 19( 1): 73– 88. doi:10.15252/embr.201744523. 29217659 PMC5757218

[39] Moudry P , Lukas C , Macurek L , Neumann B , Heriche JK , Pepperkok R , et al. Nucleoporin NUP153 guards genome integrity by promoting nuclear import of 53BP1. Cell Death Differ. 2012; 19( 5): 798– 807. doi:10.1038/cdd.2011.150. 22075984 PMC3321618

[40] Wang Z , Liu Y , Zhang Y , Shi J , Xie S , Yi M , et al. TSPYL5-driven G3BP1 nuclear membrane translocation facilitates p53 cytoplasm sequestration via accelerating RanBP2-mediated p53 sumoylation and nuclear export in neuroblastoma. Cell Death Dis. 2025; 16( 1): 358. doi:10.1038/s41419-025-07694-x. 40319028 PMC12049415

[41] Kaya F , Bewicke-Copley F , Miettinen JJ , Casado P , Leddy E , Deniz Ö , et al. DEK::NUP214 acts as an XPO1-dependent transcriptional activator of essential leukemia genes. Leukemia. 2025; 39( 6): 1526– 31. doi:10.1038/s41375-025-02593-8. 40204893 PMC12133580

[42] Oka M , Mura S , Otani M , Miyamoto Y , Nogami J , Maehara K , et al. Chromatin-bound CRM1 recruits SET-Nup214 and NPM1c onto HOX clusters causing aberrant HOX expression in leukemia cells. eLife. 2019; 8: e46667. 31755865 10.7554/eLife.46667 PMC6874418

[43] Kirthika P , Jawalagatti V , Li P , Xu M , Carceles-Cordon M , Ertel A , et al. Off-pore nucleoporin sPOM121 transcriptionally propels β-catenin-driven tumor progression and immune escape in prostate cancer. Cancer Discov. 2025; 15( 11): 2374– 96. doi:10.1158/2159-8290.CD-25-0629. 40709833 PMC12580793

[44] Celetti G , Paci G , Caria J , VanDelinder V , Bachand G , Lemke EA . The liquid state of FG-nucleoporins mimics permeability barrier properties of nuclear pore complexes. J Cell Biol. 2020; 219( 1): e201907157. 31723007 10.1083/jcb.201907157 PMC7039189

[45] Ahn JH , Davis ES , Daugird TA , Zhao S , Quiroga IY , Uryu H , et al. Phase separation drives aberrant chromatin looping and cancer development. Nature. 2021; 595( 7868): 591– 5. doi:10.1038/s41586-021-03662-5. 34163069 PMC8647409

[46] Chandra B , Michmerhuizen NL , Shirnekhi HK , Tripathi S , Pioso BJ , Baggett DW , et al. Phase Separation mediates NUP98 fusion oncoprotein leukemic transformation. Cancer Discov. 2022; 12( 4): 1152– 69. doi:10.1158/2159-8290.CD-21-0674. 34903620 PMC8983581

[47] Zhao L , Luo B , Wang L , Chen W , Jiang M , Zhang N . Pan-cancer analysis reveals the roles of XPO1 in predicting prognosis and tumorigenesis. Transl Cancer Res. 2021; 10( 11): 4664– 79. doi:10.21037/tcr-21-1646. 35116322 PMC8797940

[48] Kirtonia A , Pandya G , Singh A , Kumari R , Singh B , Kapoor S , et al. Anticancer and therapeutic efficacy of XPO1 inhibition in pancreatic ductal adenocarcinoma through DNA damage and modulation of miR-193b/KRAS/LAMC2/ERK/AKT signaling cascade. Life Sci. 2025; 362: 123364. doi:10.1016/j.lfs.2024.123364. 39778762

[49] Kruer TL , Quintana-Gonzalez A , Newman HL , Ferrall-Fairbanks MC , Zhang L , McLemore AF , et al. XPO1 drives resistance to eprenetapopt and azacitidine and can be targeted in TP53-mutated myeloid malignancies. Blood. 2025; 146( 18): 2244– 58. doi:10.1182/blood.2025028803. 40737597

[50] Corno C , Stucchi S , De Cesare M , Carenini N , Stamatakos S , Ciusani E , et al. FoxO-1 contributes to the efficacy of the combination of the XPO1 inhibitor selinexor and cisplatin in ovarian carcinoma preclinical models. Biochem Pharmacol. 2018; 147: 93– 103. doi:10.1016/j.bcp.2017.11.009. 29155058

[51] Kim E , Mordovkina DA , Sorokin A . Targeting XPO1-dependent nuclear export in cancer. Biochemistry. 2022; 87( Suppl 1): S178– 91. doi:10.1134/S0006297922140140. 35501995

[52] El-Tanani M , Dakir E-H , Raynor B , Morgan R . Mechanisms of nuclear export in cancer and resistance to chemotherapy. Cancers. 2016; 8( 3): 35. doi:10.3390/cancers8030035. 26985906 PMC4810119

[53] Ferreira CA , Schneider PN , Carneiro LT , Mendonça BS , Nestal de Moraes G . Importin α/β inhibition as a strategy to modulate cancer drug resistance and XIAP nuclear translocation. Biochem Biophys Res Commun. 2025; 751: 151409. doi:10.1016/j.bbrc.2025.151409. 39919389

[54] Huang JL , Yan XL , Huang D , Gan L , Gao H , Fan RZ , et al. Discovery of a highly potent and orally available importin-β1 inhibitor that overcomes enzalutamide-resistance in advanced prostate cancer. Acta Pharm Sin B. 2023; 13( 12): 4934– 44. doi:10.1016/j.apsb.2023.07.017. 38045040 PMC10692375

[55] Chi RPA , van der Watt P , Wei W , Birrer MJ , Leaner VD . Inhibition of Kpnβ1 mediated nuclear import enhances cisplatin chemosensitivity in cervical cancer. BMC Cancer. 2021; 21( 1): 106. doi:10.1186/s12885-021-07819-3. 33530952 PMC7852134

[56] Vercruysse T , Vanstreels E , Jacquemyn M , Boland S , Kilonda A , Allasia S , et al. Ibetazol, a novel inhibitor of importin β1-mediated nuclear import. Commun Biol. 2024; 7( 1): 1560. doi:10.1038/s42003-024-07237-8. 39580542 PMC11585640

[57] Zhou Y , Liu F , Xu Q , Yang B , Li X , Jiang S , et al. Inhibiting Importin 4-mediated nuclear import of CEBPD enhances chemosensitivity by repression of PRKDC-driven DNA damage repair in cervical cancer. Oncogene. 2020; 39( 34): 5633– 48. doi:10.1038/s41388-020-1384-3. 32661323 PMC7441007

[58] Cai GX , Kong WY , Liu Y , Zhong SY , Liu Q , Deng YF , et al. Nuclear transport maintenance of USP22-AR by Importin-7 promotes breast cancer progression. Cell Death Discov. 2023; 9( 1): 211. doi:10.1038/s41419-023-05738-8. 37391429 PMC10313651

[59] Liu A , Yu C , Qiu C , Wu Q , Huang C , Li X , et al. PRMT5 methylating SMAD4 activates TGF-β signaling and promotes colorectal cancer metastasis. Oncogene. 2023; 42( 19): 1572– 84. doi:10.1038/s41388-023-02674-x. 36991117

[60] Repenning A , Happel D , Bouchard C , Meixner M , Verel-Yilmaz Y , Raifer H , et al. PRMT1 promotes the tumor suppressor function of p14ARF and is indicative for pancreatic cancer prognosis. EMBO J. 2021; 40( 13): e106777. doi:10.15252/embj.2020106777. 33999432 PMC8246066

[61] Nakakido M , Deng Z , Suzuki T , Dohmae N , Nakamura Y , Hamamoto R . PRMT6 increases cytoplasmic localization of p21CDKN1A in cancer cells through arginine methylation and makes more resistant to cytotoxic agents. Oncotarget. 2015; 6( 31): 30957– 67. doi:10.18632/oncotarget.5143. 26436589 PMC4741580

[62] Hong XL , Huang CK , Qian H , Ding CH , Liu F , Hong HY , et al. Positive feedback between arginine methylation of YAP and methionine transporter SLC43A2 drives anticancer drug resistance. Nat Commun. 2025; 16( 1): 87. doi:10.1038/s41467-024-55769-8. 39747898 PMC11697449

[63] Hu Y , Dong Z , Liu K . Unraveling the complexity of STAT3 in cancer: Molecular understanding and drug discovery. J Exp Clin Cancer Res. 2024; 43( 1): 23. doi:10.1186/s13046-024-02949-5. 38245798 PMC10799433

[64] Geffen Y , Anand S , Akiyama Y , Yaron TM , Song Y , Johnson JL , et al. Pan-cancer analysis of post-translational modifications reveals shared patterns of protein regulation. Cell. 2023; 186( 18): 3945– 67.e26. 37582358 10.1016/j.cell.2023.07.013 PMC10680287

[65] Wan T , He M , Liu Z , Zhou Y , Zhou Y , Xiao W , et al. Phosphorylation of KIAA1429 promotes oxaliplatin resistance through activating the FZD7-Wnt signaling in BRAFV600E-mutated colorectal cancer. J Exp Clin Cancer Res. 2025; 44( 1): 187. doi:10.1186/s13046-025-03449-w. 40611274 PMC12225155

[66] Liu J , Wang H , Wan H , Yang J , Gao L , Wang Z , et al. NEK6 dampens FOXO3 nuclear translocation to stabilize C-MYC and promotes subsequent *de novo* purine synthesis to support ovarian cancer chemoresistance. Cell Death Dis. 2024; 15( 9): 661. doi:10.1038/s41419-024-07045-2. 39256367 PMC11387829

[67] Wang Z , Gu Y , Liu Y , Wang Z , Chen X , Wang H , et al. Phosphorylated Toll-like receptor 3 nuclear translocation in cancer cell promotes metastasis and chemoresistance. Signal Transduct Target Ther. 2025; 10( 1): 225. doi:10.1038/s41392-025-02307-7. 40675966 PMC12271568

[68] Lin JF , Liu ZX , Chen DL , Huang RZ , Cao F , Yu K , et al. Nucleus-translocated GCLM promotes chemoresistance in colorectal cancer through a moonlighting function. Nat Commun. 2025; 16( 1): 263. doi:10.1038/s41467-024-55568-1. 39747101 PMC11696352

[69] Tang J , Li J , Lian J , Huang Y , Zhang Y , Lu Y , et al. CDK2-activated TRIM32 phosphorylation and nuclear translocation promotes radioresistance in triple-negative breast cancer. J Adv Res. 2024; 61: 239– 51. doi:10.1016/j.jare.2023.09.011. 37734566 PMC11258662

[70] Yi Y , Li P , Huang Y , Chen D , Fan S , Wang J , et al. P21-activated kinase 2-mediated β-catenin signaling promotes cancer stemness and osimertinib resistance in EGFR-mutant non-small-cell lung cancer. Oncogene. 2022; 41( 37): 4318– 29. doi:10.1038/s41388-022-02438-z. 35986102

[71] Ma H , Qi G , Han F , Peng J , Yuan C , Kong B . PBK drives PARP inhibitor resistance through the TRIM37/NFκB axis in ovarian cancer. Exp Mol Med. 2022; 54( 7): 999– 1010. doi:10.1038/s12276-022-00809-w. 35859118 PMC9355941

[72] Kang T , Wang Y , Jiang Y , Chen S , Lin N , Guo M , et al. The SGK3/GSK3β/β-catenin signaling promotes breast cancer stemness and confers resistance to alpelisib therapy. Int J Biol Sci. 2025; 21( 6): 2462– 75. doi:10.7150/ijbs.104850. 40303291 PMC12035905

[73] Seeler JS , Dejean A . SUMO and the robustness of cancer. Nat Rev Cancer. 2017; 17( 3): 184– 97. doi:10.1038/nrc.2016.143. 28134258

[74] Rallabhandi P , Hashimoto K , Mo YY , Beck WT , Moitra PK , D’Arpa P . Sumoylation of topoisomerase I is involved in its partitioning between nucleoli and nucleoplasm and its clearing from nucleoli in response to camptothecin. J Biol Chem. 2002; 277( 42): 40020– 6. doi:10.1074/jbc.M200388200. 12149243

[75] Jiang K , Yang W , Huang J , Tan X , Liu Y , Tu S , et al. SENP1 promotes p27kip1 nuclear export though enhanced SUMOylation in cholangiocarcinoma leading to increased cell proliferation and chemoresistance. Int J Mol Med. 2025; 56( 3): 141. doi:10.3892/ijmm.2025.5582. 40682835 PMC12270383

[76] Zheng S , Tian Q , Yuan Y , Sun S , Li T , Xia R , et al. Extracellular vesicle-packaged circBIRC6 from cancer-associated fibroblasts induce platinum resistance via SUMOylation modulation in pancreatic cancer. J Exp Clin Cancer Res. 2023; 42( 1): 324. doi:10.1186/s13046-023-02854-3. 38012734 PMC10683239

[77] Gao H , Xi Z , Dai J , Xue J , Guan X , Zhao L , et al. Drug resistance mechanisms and treatment strategies mediated by Ubiquitin-Specific Proteases (USPs) in cancers: New directions and therapeutic options. Mol Cancer. 2024; 23( 1): 88. doi:10.1186/s12943-024-02005-y. 38702734 PMC11067278

[78] Wei J , Wang L , Zhang Y , Sun T , Zhang C , Hu Z , et al. TRIM25 promotes temozolomide resistance in glioma by regulating oxidative stress and ferroptotic cell death via the ubiquitination of keap1. Oncogene. 2023; 42( 26): 2103– 12. doi:10.1038/s41388-023-02717-3. 37188737

[79] He M , Zhou X , Wang X . Glycosylation: Mechanisms, biological functions and clinical implications. Signal Transduct Target Ther. 2024; 9( 1): 194. doi:10.1038/s41392-024-01886-1. 39098853 PMC11298558

[80] Very N , Lefebvre T , El Yazidi-Belkoura I . Drug resistance related to aberrant glycosylation in colorectal cancer. Oncotarget. 2017; 9( 1): 1380– 402. doi:10.18632/oncotarget.22377. 29416702 PMC5787446

[81] Honma K , Iwao-Koizumi K , Takeshita F , Yamamoto Y , Yoshida T , Nishio K , et al. RPN2 gene confers docetaxel resistance in breast cancer. Nat Med. 2008; 14( 9): 939– 48. doi:10.1038/nm.1858. 18724378

[82] Zhang Y , Zhou S , Kai Y , Zhang YQ , Peng C , Li Z , et al. O-GlcNAcylation of MITF regulates its activity and CDK4/6 inhibitor resistance in breast cancer. Nat Commun. 2024; 15( 1): 5597. doi:10.1038/s41467-024-49875-w. 38961064 PMC11222436

[83] Wang Y , Su K , Wang C , Deng T , Liu X , Sun S , et al. Chemotherapy-induced acetylation of ACLY by NAT10 promotes its nuclear accumulation and acetyl-CoA production to drive chemoresistance in hepatocellular carcinoma. Cell Death Dis. 2024; 15( 7): 545. doi:10.1038/s41419-024-06951-9. 39085201 PMC11291975

[84] Dutta H , Jain N . Post-translational modifications and their implications in cancer. Front Oncol. 2023; 13: 1240115. doi:10.3389/fonc.2023.1240115. 37795435 PMC10546021

[85] Gounder MM , Martin-Romano P , Italiano A , Siu LL , Cassier PA , Falchook GS , et al. Phase 1b and Dose-expansion study of GSK3326595, a PRMT5 inhibitor as monotherapy and in combination with pembrolizumab in patients with advanced cancers. Ann Oncol. 2025; 36( 12): 1480– 91. doi:10.1016/j.annonc.2025.08.3757. 40935292

[86] Li Y , Seto E . HDACs and HDAC Inhibitors in Cancer Development and Therapy. Cold Spring Harb Perspect Med. 2016; 6( 10): a026831. doi:10.1101/cshperspect.a026831. 27599530 PMC5046688

[87] Groelly FJ , Fawkes M , Dagg RA , Blackford AN , Tarsounas M . Targeting DNA damage response pathways in cancer. Nat Rev Cancer. 2023; 23( 2): 78– 94. doi:10.1038/s41568-022-00535-5. 36471053

[88] Di Y , Zhang X , Wen X , Qin J , Ye L , Wang Y , et al. MAPK signaling-mediated RFNG phosphorylation and nuclear translocation restrain oxaliplatin-induced apoptosis and ferroptosis. Adv Sci. 2024; 11( 38): 2402795. doi:10.1002/advs.202402795. PMC11481204 39120977

[89] Myatt SS , Lam EWF . The emerging roles of forkhead box (Fox) proteins in cancer. Nat Rev Cancer. 2007; 7( 11): 847– 59. doi:10.1038/nrc2223. 17943136

[90] Gomes AR , Brosens JJ , Lam EWF . Resist or die: FOXO transcription factors determine the cellular response to chemotherapy. Cell Cycle. 2008; 7( 20): 3133– 6. doi:10.4161/cc.7.20.6920. 18927504

[91] Kashyap T , Argueta C , Aboukameel A , Unger TJ , Klebanov B , Mohammad RM , et al. Selinexor, a Selective Inhibitor of Nuclear Export (SINE) compound, acts through NF-κB deactivation and combines with proteasome inhibitors to synergistically induce tumor cell death. Oncotarget. 2016; 7( 48): 78883– 95. doi:10.18632/oncotarget.12428. 27713151 PMC5346685

[92] Zhu X , Shen H , Yin X , Long L , Chen X , Feng F , et al. IL-6R/STAT3/miR-204 feedback loop contributes to cisplatin resistance of epithelial ovarian cancer cells. Oncotarget. 2017; 8( 24): 39154– 66. doi:10.18632/oncotarget.16610. 28388577 PMC5503602

[93] Kofler M , Kapus A . Nuclear import and export of YAP and TAZ. Cancers. 2023; 15( 20): 4956. doi:10.3390/cancers15204956. 37894323 PMC10605228

[94] Qian H , Ding CH , Liu F , Chen SJ , Huang CK , Xiao MC , et al. SRY-Box transcription factor 9 triggers YAP nuclear entry via direct interaction in tumors. Signal Transduct Target Ther. 2024; 9( 1): 96. doi:10.1038/s41392-024-01805-4. 38653754 PMC11039692

[95] Chang HA , Ou Yang RZ , Su JM , Nguyen TMH , Sung JM , Tang MJ , et al. YAP nuclear translocation induced by HIF-1α prevents DNA damage under hypoxic conditions. Cell Death Discov. 2023; 9( 1): 385. doi:10.1038/s41420-023-01687-5. 37863897 PMC10589224

[96] Nie L , Wang YN , Hsu JM , Hou J , Chu YY , Chan LC , et al. Nuclear export signal mutation of epidermal growth factor receptor enhances malignant phenotypes of cancer cells. Am J Cancer Res. 2023; 13( 4): 1209– 39. 37168336 PMC10164793

[97] Mirski SE , Evans CD , Almquist KC , Slovak ML , Cole SP . Altered topoisomerase II alpha in a drug-resistant small cell lung cancer cell line selected in VP-16. Cancer Res. 1993; 53( 20): 4866– 73. 8104687

[98] Reich TR , Schwarzenbach C , Vilar JB , Unger S , Mühlhäusler F , Nikolova T , et al. Localization matters: Nuclear-trapped Survivin sensitizes glioblastoma cells to temozolomide by elevating cellular senescence and impairing homologous recombination. Cell Mol Life Sci. 2021; 78( 14): 5587– 604. doi:10.1007/s00018-021-03864-0. 34100981 PMC8257519

[99] Zhao H , Faltermeier CM , Mendelsohn L , Porter PL , Clurman BE , Roberts JM . Mislocalization of p27 to the cytoplasm of breast cancer cells confers resistance to anti-HER2 targeted therapy. Oncotarget. 2014; 5( 24): 12704– 14. doi:10.18632/oncotarget.2871. 25587029 PMC4350358

[100] Dai XY , Zhuang LH , Wang DD , Zhou TY , Chang LL , Gai RH , et al. Nuclear translocation and activation of YAP by hypoxia contributes to the chemoresistance of SN38 in hepatocellular carcinoma cells. Oncotarget. 2016; 7( 6): 6933– 47. doi:10.18632/oncotarget.6903. 26771844 PMC4872759

[101] Xiong H , Nie X , Zou Y , Gong C , Li Y , Wu H , et al. Twist1 enhances hypoxia induced radioresistance in cervical cancer cells by promoting nuclear EGFR localization. J Cancer. 2017; 8( 3): 345– 53. doi:10.7150/jca.16607. 28261334 PMC5332884

[102] Saieva L , Barreca MM , Zichittella C , Prado MG , Tripodi M , Alessandro R , et al. Hypoxia-induced miR-675-5p supports β-catenin nuclear localization by regulating GSK3-β Activity in colorectal cancer cell lines. Int J Mol Sci. 2020; 21( 11): 3832. doi:10.3390/ijms21113832. 32481626 PMC7312749

[103] Syu JP , Chi JT , Kung HN . Nrf2 is the key to chemotherapy resistance in MCF7 breast cancer cells under hypoxia. Oncotarget. 2016; 7( 12): 14659– 72. doi:10.18632/oncotarget.7406. 26894974 PMC4924742

[104] Joyce MH , Lu C , James ER , Hegab R , Allen SC , Suggs LJ , et al. Phenotypic basis for matrix stiffness-dependent chemoresistance of breast cancer cells to doxorubicin. Front Oncol. 2018; 8: 337. doi:10.3389/fonc.2018.00337. 30234012 PMC6134055

[105] Rice AJ , Cortes E , Lachowski D , Cheung BCH , Karim SA , Morton JP , et al. Matrix stiffness induces epithelial-mesenchymal transition and promotes chemoresistance in pancreatic cancer cells. Oncogenesis. 2017; 6( 7): e352. 28671675 10.1038/oncsis.2017.54 PMC5541706

[106] Yang L , Kono T , Gilbertsen A , Li Y , Sun B , Jacobson BA , et al. GPR81 nuclear transportation is critical for cancer growth and progression in lung and other solid cancers. World J Clin Oncol. 2025; 16( 8): 107208. doi:10.5306/wjco.v16.i8.107208. 40901330 PMC12400187

[107] Newell S , van der Watt PJ , Leaner VD . Therapeutic targeting of nuclear export and import receptors in cancer and their potential in combination chemotherapy. IUBMB Life. 2023; 76( 1): 4– 25. doi:10.1002/iub.2773. 37623925 PMC10952567

[108] He Y , Wang Q , Wang Z , Duan M , Zhou Y , Huang J , et al. The functional and clinical significance of nucleoporin NUP153 across human cancers: A systematic study based on multi-omics analysis and bench work validation. Front Immunol. 2025; 16: 1613688. doi:10.3389/fimmu.2025.1613688. 40607419 PMC12213620

[109] Sakuma S , Raices M , Borlido J , Guglielmi V , Zhu EYS , D’Angelo MA . Inhibition of nuclear pore complex formation selectively induces cancer cell death. Cancer Discov. 2021; 11( 1): 176– 93. doi:10.1158/2159-8290.CD-20-0581. 32988961 PMC8004544

[110] González-Calle V , Rodríguez-Otero P , Sureda A , De Arriba F , Reinoso M , Ribas P , et al. Selinexor, daratumumab, bortezomib and dexamethasone for the treatment of patients with relapsed or refractory multiple myeloma: Results of the phase II, nonrandomized, multicenter GEM-SELIBORDARA study. Haematologica. 2024; 109( 7): 2219– 28. doi:10.3324/haematol.2023.284089. 38356463 PMC11215366

[111] Nataraj NB , Noronha A , Lee JS , Ghosh S , Mohan Raju HR , Sekar A , et al. Nucleoporin-93 reveals a common feature of aggressive breast cancers: Robust nucleocytoplasmic transport of transcription factors. Cell Rep. 2022; 38( 8): 110418. doi:10.1016/j.celrep.2022.110418. 35196484 PMC8957480

[112] Hong X , Li Q , Li J , Chen K , He Q , Zhao Y , et al. CircIPO7 promotes nasopharyngeal carcinoma metastasis and cisplatin chemoresistance by facilitating YBX1 nuclear localization. Clin Cancer Res. 2022; 28( 20): 4521– 35. doi:10.1158/1078-0432.CCR-22-0991. 35917517

[113] Wang JY , Chen MP , Jiang JX , Wan YK , Li X , Zhang YW , et al. Lipin1-dependent transcriptional inactivation of SREBPs contributes to selinexor sensitivity in multiple myeloma. Acta Pharmacol Sin. 2025; 46( 9): 2496– 508. doi:10.1038/s41401-025-01553-3. 40229499 PMC12373733

[114] Meng M , Feng X , Zhang Y , Gao Y , Han L , Li Z , et al. Efficacy and mechanism of the XPO1 inhibitor selinexor combined with decitabine in T-cell lymphoblastic lymphoma. Ann Hematol. 2025; 104( 3): 1747– 56. doi:10.1007/s00277-025-06271-8. 40014082 PMC12031777

[115] Xu J , Wu S , Li G . Selective nuclear export inhibitor KPT-330 enhances the radiosensitivity of esophageal carcinoma cells. Exp Ther Med. 2023; 26( 1): 326. doi:10.3892/etm.2023.12025. 37346402 PMC10280315

[116] Jayashankar V , Kubiniok P , McCracken AN , Gentry RG , Eckenstein KH , Sernissi L , et al. Sphingosine simultaneously inhibits nuclear import and activates PP2A by binding importins and PPP2R1A. EMBO J. 2025; 44( 16): 4473– 98. doi:10.1038/s44318-025-00490-5. 40588551 PMC12361511

[117] Yu L , Deng Y , Wang X , Santos C , Davis IJ , Earp HS , et al. Co-targeting JAK1/STAT6/GAS6/TAM signaling improves chemotherapy efficacy in Ewing sarcoma. Nat Commun. 2024; 15( 1): 5292. doi:10.1038/s41467-024-49667-2. 38906855 PMC11192891

[118] Emdal KB , Palacio-Escat N , Wigerup C , Eguchi A , Nilsson H , Bekker-Jensen DB , et al. Phosphoproteomics of primary AML patient samples reveals rationale for AKT combination therapy and p53 context to overcome selinexor resistance. Cell Rep. 2022; 40( 6): 111177. doi:10.1016/j.celrep.2022.111177. 35947955 PMC9380259

[119] Liu M , Xu C , Qin X , Liu W , Li D , Jia H , et al. DHW-221, a Dual PI3K/mTOR Inhibitor, Overcomes Multidrug Resistance by Targeting P-Glycoprotein (P-gp/ABCB1) and Akt-Mediated FOXO3a Nuclear Translocation in Non-small Cell Lung Cancer. Front Oncol. 2022; 12: 873649. doi:10.3389/fonc.2022.873649. 35646704 PMC9137409

[120] Mittal S , Kadamberi IP , Chang H , Wang F , Kumar S , Tsaih S-W , et al. Preclinical activity of selinexor in combination with eribulin in uterine leiomyosarcoma. Exp Hematol Oncol. 2023; 12( 1): 78. doi:10.1186/s40164-023-00443-w. 37715291 PMC10503035

[121] Paulson AL , Gruener RF , Lee AM , Huang RS . Discovery, validation and mechanistic study of XPO1 Inhibition in the treatment of triple-negative breast cancer. Cancers. 2024; 16( 23): 3980. doi:10.3390/cancers16233980. 39682167 PMC11640544

[122] Li FL , Liu JP , Bao RX , Yan G , Feng X , Xu YP , et al. Acetylation accumulates PFKFB3 in cytoplasm to promote glycolysis and protects cells from cisplatin-induced apoptosis. Nat Commun. 2018; 9( 1): 508. doi:10.1038/s41467-018-02950-5. 29410405 PMC5802808

[123] Song J , Yang P , Chen C , Ding W , Tillement O , Bai H , et al. Targeting epigenetic regulators as a promising avenue to overcome cancer therapy resistance. Signal Transduct Target Ther. 2025; 10( 1): 219. doi:10.1038/s41392-025-02266-z. 40675967 PMC12271501

[124] El-Khoueiry AB , Clarke J , Neff T , Crossman T , Ratia N , Rathi C , et al. Phase 1 study of GSK3368715, a type I PRMT inhibitor, in patients with advanced solid tumors. Br J Cancer. 2023; 129( 2): 309– 17. doi:10.1038/s41416-023-02276-0. 37237172 PMC10338470

[125] Zhu Y , Xia T , Chen DQ , Xiong X , Shi L , Zuo Y , et al. Promising role of protein arginine methyltransferases in overcoming anti-cancer drug resistance. Drug Resist Updat. 2023; 72: 101016. doi:10.1016/j.drup.2023.101016. 37980859

[126] Kaganovski A , Smith-Salzberg B , Shimshon HK , Draheim A , Spivak M , Sapir T , et al. Current and emerging therapies for targeting protein arginine methyltransferases (PRMTs) in cancer. Int J Mol Sci. 2025; 26( 16): 7907. doi:10.3390/ijms26167907. 40869229 PMC12386604

[127] Okabe S , Arai Y , Tanaka Y , Gotoh A . Therapeutic potential of venetoclax and selinexor in targeting hypoxia-induced vulnerabilities in multiple myeloma. Cancer Rep. 2025; 8( 6): e70249. doi:10.1002/cnr2.70249. PMC12180287 40539846

[128] Xu K , Zhan Y , Yuan Z , Qiu Y , Wang H , Fan G , et al. Hypoxia Induces drug resistance in colorectal cancer through the HIF-1α/miR-338-5p/IL-6 feedback loop. Mol Ther. 2019; 27( 10): 1810– 24. doi:10.1016/j.ymthe.2019.05.017. 31208913 PMC6822233

[129] Zhu ZJ , Pang Y , Jin G , Zhang HY , Wang WH , Liu JW , et al. Hypoxia induces chemoresistance of esophageal cancer cells to cisplatin through regulating the lncRNA-EMS/miR-758-3p/WTAP axis. Aging. 2021; 13( 13): 17155– 76. doi:10.18632/aging.203062. 34081626 PMC8312407

[130] Schwartz DL , Powis G , Thitai-Kumar A , He Y , Bankson J , Williams R , et al. The selective hypoxia inducible factor-1 inhibitor PX-478 provides *in vivo* radiosensitization through tumor stromal effects. Mol Cancer Ther. 2009; 8( 4): 947– 58. doi:10.1158/1535-7163.MCT-08-0981. 19372568 PMC2908257

[131] Faes S , Duval AP , Planche A , Uldry E , Santoro T , Pythoud C , et al. Acidic tumor microenvironment abrogates the efficacy of mTORC1 inhibitors. Mol Cancer. 2016; 15( 1): 78. doi:10.1186/s12943-016-0562-y. 27919264 PMC5139076

[132] Ronca R , Supuran CT . Carbonic anhydrase IX: An atypical target for innovative therapies in cancer. Biochim Biophys Acta Rev Cancer. 2024; 1879( 4): 189120. doi:10.1016/j.bbcan.2024.189120. 38801961

[133] Andreucci E , Biagioni A , Peri S , Versienti G , Cianchi F , Staderini F , et al. The CAIX inhibitor SLC-0111 exerts anti-cancer activity on gastric cancer cell lines and resensitizes resistant cells to 5-Fluorouracil, taxane-derived, and platinum-based drugs. Cancer Lett. 2023; 571: 216338. doi:10.1016/j.canlet.2023.216338. 37549770

[134] Chafe SC , McDonald PC , Saberi S , Nemirovsky O , Venkateswaran G , Burugu S , et al. Targeting hypoxia-induced carbonic anhydrase IX enhances immune-checkpoint blockade locally and systemically. Cancer Immunol Res. 2019; 7( 7): 1064– 78. doi:10.1158/2326-6066.CIR-18-0657. 31088846

[135] Curtis NJ , Mooney L , Hopcroft L , Michopoulos F , Whalley N , Zhong H , et al. Pre-clinical pharmacology of AZD3965, a selective inhibitor of MCT1: DLBCL, NHL and Burkitt’s lymphoma anti-tumor activity. Oncotarget. 2017; 8( 41): 69219– 36. doi:10.18632/oncotarget.18215. 29050199 PMC5642474

[136] Preyer M , Vigneri P , Wang JYJ . Interplay between kinase domain autophosphorylation and F-actin binding domain in regulating imatinib sensitivity and nuclear import of BCR-ABL. PLoS One. 2011; 6( 2): e17020. 21347248 10.1371/journal.pone.0017020 PMC3037956

[137] Li Y-F , Pan X , Shen H-B . Discovering the nuclear localization signal universe through a deep learning model with interpretable attention units. Patterns. 2025; 6( 6): 101262. doi:10.1016/j.patter.2025.101262. 40575124 PMC12191761

[138] Xu D , Grishin NV , Chook YM . NESdb: A database of NES-containing CRM1 cargoes. Mol Biol Cell. 2012; 23( 18): 3673– 6. doi:10.1091/mbc.e12-01-0045. 22833564 PMC3442414

[139] Kosugi S , Yanagawa H , Terauchi R , Tabata S . NESmapper: Accurate prediction of leucine-rich nuclear export signals using activity-based profiles. PLoS Comput Biol. 2014; 10( 9): e1003841. doi:10.1371/journal.pcbi.1003841. 25233087 PMC4168985

[140] Lee Y , Baumhardt JM , Pei J , Chook YM , Grishin NV . pCRM1exportome: Database of predicted CRM1-dependent Nuclear Export Signal (NES) motifs in cancer-related genes. Bioinformatics. 2020; 36( 3): 961– 3. doi:10.1093/bioinformatics/btz657. 31504173 PMC8496372

[141] Peterson TJ , Orozco J , Buege M . Selinexor: A first-in-class nuclear export inhibitor for management of multiply relapsed multiple myeloma. Ann Pharmacother. 2019; 54( 6): 577– 82. doi:10.1177/1060028019892643. 31793336 PMC8498942

[142] Derman BA , Chari A , Zonder J , Major A , Stefka AT , Jiang K , et al. A phase I study of selinexor combined with weekly carfilzomib and dexamethasone in relapsed/refractory multiple myeloma. Eur J Haematol. 2023; 110( 5): 564– 70. doi:10.1111/ejh.13937. 36726221

[143] Maerevoet M , Zijlstra JM , Follows G , Casasnovas RO , Vermaat JSP , Kalakonda N , et al. Survival among patients with relapsed/refractory diffuse large B cell lymphoma treated with single-agent selinexor in the SADAL study. J Hematol Oncol. 2021; 14( 1): 111. doi:10.1186/s13045-021-01122-1. 34271963 PMC8283921

[144] Mascarenhas J , Maher K , Rampal R , Bose P , Podoltsev N , Hong J , et al. Selinexor plus ruxolitinib in JAK inhibitor treatment-naïve myelofibrosis: SENTRY Phase 3 study design. Future Oncol. 2025; 21( 7): 807– 13. doi:10.1080/14796694.2025.2461393. 39911057 PMC11916360

[145] Ball S , Awan FT , Tomlinson BK , Stopczynski T , Fischer MA , Zhao Z , et al. Selinexor and venetoclax combination in patients with relapsed or refractory acute myeloid leukemia. Am J Hematol. 2026; 101( 5): 1019– 24. doi:10.1002/ajh.70266. 41796974 PMC13055113

[146] Gounder MM , Zer A , Tap WD , Salah S , Dickson MA , Gupta AA , et al. Phase IB study of selinexor, a first-in-class inhibitor of nuclear export, in patients with advanced refractory bone or soft tissue sarcoma. J Clin Oncol. 2016; 34( 26): 3166– 74. doi:10.1200/JCO.2016.67.6346. 27458288 PMC5321073

[147] Green AL , Minard CG , Liu X , Safgren SL , Pinkney K , Harris L , et al. Phase I trial of selinexor in pediatric recurrent/refractory solid and CNS tumors (ADVL1414): A children’s oncology group phase I consortium trial. Clin Cancer Res. 2025; 31( 9): 1587– 95. doi:10.1158/1078-0432.CCR-24-2754. 39998852 PMC12045713

[148] Lassman AB , Wen PY , van den Bent MJ , Plotkin SR , Walenkamp AME , Green AL , et al. A phase II Study of the efficacy and safety of oral selinexor in recurrent glioblastoma. Clin Cancer Res. 2021; 28( 3): 452– 60. doi:10.1158/1078-0432.CCR-21-2225. 34728525 PMC8810630

[149] Hernando-Calvo A , Malone E , Day D , Prawira A , Weinreb I , Yang SYC , et al. Selinexor for the treatment of recurrent or metastatic salivary gland tumors: Results from the GEMS-001 clinical trial. Cancer Med. 2023; 12( 20): 20299– 310. doi:10.1002/cam4.6589. 37818869 PMC10652322

[150] Thein KZ , Piha-Paul SA , Tsimberidou A , Karp DD , Janku F , Fu S , et al. Selinexor in combination with standard chemotherapy in patients with advanced or metastatic solid tumors. Exp Hematol Oncol. 2021; 10( 1): 59. doi:10.1186/s40164-021-00251-0. 34965890 PMC8715578

[151] von Itzstein MS , Burns TF , Dowell JE , Horn L , Camidge DR , York SJ , et al. Phase I/II trial of exportin 1 inhibitor Selinexor plus docetaxel in previously treated, advanced KRAS-Mutant non-small cell lung cancer. Clin Cancer Res. 2025; 31( 4): 639– 48. doi:10.1158/1078-0432.CCR-24-1722. 39651955 PMC11832340

[152] Liu Y , Yun X , Ding W , Li S , Liu H . Targeting the nuclear export receptor exportin-1 in acute myeloid leukaemia: From biology to clinical translation. Clin Transl Med. 2026; 16( 5): e70676. doi:10.1002/ctm2.70676. 42071259 PMC13136070

[153] Neggers JE , Vanstreels E , Baloglu E , Shacham S , Landesman Y , Daelemans D . Heterozygous mutation of cysteine528 in XPO1 is sufficient for resistance to selective inhibitors of nuclear export. Oncotarget. 2016; 7( 42): 68842– 50. doi:10.18632/oncotarget.11995. 27634897 PMC5356594

[154] Lee S , Mohan S , Knupp J , Chamoun K , de Jonge A , Yang F , et al. Oral eltanexor treatment of patients with higher-risk myelodysplastic syndrome refractory to hypomethylating agents. J Hematol Oncol. 2022; 15( 1): 103. doi:10.1186/s13045-022-01319-y. 35922861 PMC9351096

[155] Cornell RF , Baz R , Richter JR , Rossi A , Vogl DT , Chen C , et al. A phase 1 clinical trial of oral eltanexor in patients with relapsed or refractory multiple myeloma. Am J Hematol. 2022; 97( 2): E54– 8. 34817872 10.1002/ajh.26420

[156] Institute NC . Eltanexor and venetoclax for the treatment of relapsed or refractory myelodysplastic syndrome and acute myeloid leukemia. 2024. [cited 2026 May 26] Available from: https://www.cancer.gov/research/participate/clinical-trials-search/v?id=NCI-2024-03343.

[157] Razak A , Mahipal A , Diamond JR , Ribas A , Berlin JD , Azmi AS , et al. First-in-human phase I study of KPT-9274, a first-in-class dual inhibitor of PAK4 and NAMPT, in patients with advanced solid malignancies. Target Oncol. 2026; 21( 2): 187– 98. doi:10.1007/s11523-026-01206-3. 41824279 PMC13031219

[158] Cordover E , Wei J , Patel C , Shan NL , Gionco J , Sargsyan D , et al. KPT-9274, an inhibitor of PAK4 and NAMPT, Leads to downregulation of mTORC2 in triple negative breast cancer cells. Chem Res Toxicol. 2020; 33( 2): 482– 91. doi:10.1021/acs.chemrestox.9b00376. 31876149 PMC9316853

[159] Aboukameel A , Muqbil I , Senapedis W , Baloglu E , Landesman Y , Shacham S , et al. Novel p21-activated kinase 4 (PAK4) allosteric modulators overcome drug resistance and stemness in pancreatic ductal adenocarcinoma. Mol Cancer Ther. 2017; 16( 1): 76– 87. doi:10.1158/1535-7163.MCT-16-0205. 28062705 PMC5221563

[160] Mpilla GB , Uddin MH , Al-Hallak MN , Aboukameel A , Li Y , Kim SH , et al. PAK4-NAMPT dual inhibition sensitizes pancreatic neuroendocrine tumors to everolimus. Mol Cancer Ther. 2021; 20( 10): 1836– 45. doi:10.1158/1535-7163.MCT-20-1105. 34253597 PMC8492493

