# Supplementary material for: Nuclear–Cytoplasmic Axis in Cancer: From Protein Mislocalization to Anticancer Drug Resistance
Source: Oncol Res. 2026 Aug 13;34(9):11. doi: 10.32604/or.2026.083902 (PMC13494494; doi:10.32604/or.2026.083902)
Supplement: Supplementary file 1 [file OncolRes-34-83902-s001.zip › TSP_OR_83902-s001/TSP_OR_83902-s001.docx]

**Table S1:** NCT–signaling crosstalk driving therapy resistance.

| **Signaling pathway** | **Key Factor & Modification** | **NCT Alteration** | **Cancer Context** | **Drug Resistance Phenotype** | **Ref.** |
| --- | --- | --- | --- | --- | --- |
| Innate immunity/JAK–STAT | TLR3 (Ser155, JAK1 phosphorylation) | Importin-α5–mediated nuclear import; complex with PRMT5/c-Myc | Pancreatic cancer | Chemoresistance (cytotoxic agents) | [67] |
| WNT/β-catenin | KIAA1429 (BRAF phosphorylation) | Cytoplasmic retention; FZD7 stabilization | Colorectal cancer | Oxaliplatin resistance | [65] |
|  | β-catenin (PAK2 phosphorylation) | Nuclear accumulation | EGFR-mutant NSCLC | Osimertinib resistance | [70] |
|  | β-catenin/GSK3β (SGK3 phosphorylation) | β-catenin nuclear enrichment | HR+ breast cancer | PI3Kα inhibitor resistance | [72] |
| NF-κB pathway | GCLM (Thr17, p38-MAPK phosphorylation) | Importin-α5–mediated nuclear import; ↑NKRF interaction | Colorectal cancer | Oxaliplatin resistance | [68] |
|  | TRIM37 (PBK phosphorylation) | Nuclear translocation; activates NF-κB | Ovarian cancer | PARP inhibitor resistance | [71] |
| Redox/antioxidant | NRF2 (via TRIM25–Keap1 ubiquitination) | Nuclear retention of NRF2 | Glioma | Temozolomide resistance | [78] |
| ERK pathway | RFNG; ERK-mediated phosphorylation at Ser255 | Phosphorylation initiates RFNG nuclear entry and suppresses p53 activity | Colorectal cancer | Oxaliplatin resistance | [88] |
| Hypoxia–Hippo/YAP crosstalk | HIF-1α–YAP cooperation under hypoxia | Hypoxic stress promotes YAP nuclear entry | Hypoxic tumor context | Resistance to DNA damage–inducible apoptosis | [95] |
| STAT3 pathway | STAT3; phosphorylation- and acetylation-dependent regulation | Post-translational modification controls STAT3 nucleocytoplasmic localization and nuclear activity | Various cancers | Therapy resistance and immune evasion | [92] |

**Abbreviations:** NCT, nucleocytoplasmic trafficking; RFNG, RFNG O-fucosylpeptide 3-beta-N-acetylglucosaminyltransferase; PTM, post-translational modification; YAP, Yes-associated protein; TAZ, transcriptional coactivator with PDZ-binding motif; HIF-1α, hypoxia-inducible factor 1 alpha; STAT3, signal transducer and activator of transcription 3.

**Table S2:** Representative examples of NLS/NES mutations that alter protein localization and modulate anticancer drug responses.

| **Protein** | **Mutation Type/Model** | **Effect on Localization** | **Drug Type/Context** | **Phenotypic Outcome (Drug Response)** | **Reference (PMID/Source)** |
| --- | --- | --- | --- | --- | --- |
| EGFR | Hotspot NES mutations L747S / L747P (in patients and cell lines) | Impaired nuclear export → nuclear accumulation of EGFR | EGFR-TKIs (gefitinib, erlotinib, osimertinib) | Decreased TKI sensitivity / acquired resistance | [96] |
| Topoisomerase IIα (TOP2A) | C-terminal truncation eliminating tripartite NLS (H209/V6 and INER-37 models) | Cytoplasmic retention of truncated Topo IIα | Topo II inhibitors (VP-16, etoposide) | Distinct mechanism of drug resistance | [97] |
| Survivin (BIRC5) | NES point mutation (disrupting CRM1 binding) | Nuclear retention of Survivin | Radiotherapy / Temozolomide (T M Z) | Increased treatment sensitivity (“reverse resistance”) | [98] |
| p27^Kip1 (CDKN1B) | Experimental ΔNLS construct | Cytoplasmic enrichment of p27 | HER-family targeted therapy (lapatinib etc.) | Reduced apoptosis and drug sensitivity | [99] |
| BRCA1 | Engineered NLS-BRCA1 and NES-BRCA1 localization mutants | Forced nuclear vs. cytoplasmic distribution | Platinum compounds / PARP inhibitor (ABT-888) | Altered DNA repair competence and drug response | [30] |

**Abbreviations:** EGFR, epidermal growth factor receptor; NES, nuclear export signal; NLS, nuclear localization signal; EGFR-TKIs, EGFR tyrosine kinase inhibitors; TOP2A, topoisomerase IIα; VP-16, etoposide; BIRC5, survivin; TMZ, temozolomide; CDKN1B, cyclin-dependent kinase inhibitor 1B; BRCA1, breast cancer susceptibility gene 1; PARP, poly(ADP-ribose) polymerase; ABT-888, veliparib.

**Table S3:** Representative studies linking TME pressures to NCT-dependent therapeutic resistance.

| **TME Factor** | **NCT/Subcellular Localization Change** | **Affected Molecule(s)** | **Drug/Therapy Response** | **Cancer Model(s)** | **Reference(s)** |
| --- | --- | --- | --- | --- | --- |
| Hypoxia | Promotes nuclear translocation and activation of YAP; reversible by mevalonate-pathway inhibition | YAP | Confers resistance to SN38 (topoisomerase-I inhibitor); YAP–mevalonate inhibition restores chemosensitivity | Hepatocellular carcinoma (HCC) | [100] |
|  | Increases nuclear accumulation of EGFR and DNA-PKcs via Twist1 induction | EGFR, DNA-PKcs | Enhances DNA repair and radioresistance; pathway blockade re-sensitizes tumors | Cervical carcinoma | [101] |
|  | Stabilizes nuclear localization of β-catenin through H19-derived miR-675-5p | β-Catenin | Drives 5-fluorouracil (5-FU) resistance; inhibition of miR-675-5p reverses phenotype | Colorectal cancer | [102] |
|  | Activates nuclear NRF2 signaling, strengthening antioxidant capacity | NRF2 | Reduces cisplatin efficacy via enhanced redox defense | Breast-cancer models | [103] |
| ECM stiffening (mechanical pressure) | Increases nuclear enrichment of YAP/TAZ (±β-catenin) under elevated matrix rigidity | YAP, TAZ, β-Catenin | Promotes doxorubicin resistance; similar nuclear YAP activation underlies gemcitabine tolerance | 3D breast-cancer spheroids; pancreatic cancer | [104，105] |
| Lactate-rich / hypoxic microenvironment | Triggers nuclear import of GPR81 (HCAR1) under metabolic stress | GPR81 | Mechanistic evidence of atypical nuclear trafficking; drug-response link pending | Lung cancer; Breast cancer; Colorectal cancer; Glioma; Pancreatic cancer. | [106] |

**Abbreviations:** TME, tumor microenvironment; NCT, nucleocytoplasmic trafficking; YAP, Yes-associated protein; EGFR, epidermal growth factor receptor; DNA-PKcs, DNA-dependent protein kinase catalytic subunit; HCC, hepatocellular carcinoma; NRF2, nuclear factor erythroid 2-related factor 2; XPO1, exportin 1; ECM, extracellular matrix; TAZ, transcriptional co-activator with PDZ-binding motif; GPR81/HCAR1, hydroxycarboxylic acid receptor 1; SN38, 7-ethyl-10-hydroxycamptothecin; 5-FU, 5-fluorouracil.
